# Prevalence of depression, anxiety, stress, and suicide tendency among individual with long-COVID and determinants: A systematic review and meta-analysis

**DOI:** 10.1371/journal.pone.0312351

**Published:** 2025-01-28

**Authors:** Razieh Bidhendi-Yarandi, Akbar Biglarian, Jannike Lie Karlstad, Cathrine Fredriksen Moe, Enayatollah Bakhshi, Mohammad-Reza Khodaei-Ardakani, Samira Behboudi-Gandevani

**Affiliations:** 1 Psychosis Research Center, University of Social Welfare and Rehabilitation Sciences, Tehran, Iran; 2 Department of Biostatistics and Epidemiology, School of Social Health, University of Social Welfare and Rehabilitation Sciences, Tehran, Iran; 3 Social Determinants of Health Research Center, University of Social Welfare and Rehabilitation Sciences, Tehran, Iran; 4 Faculty of Nursing and Health Sciences, Nord University, Bodø, Norway; 5 Razi Psychiatric Hospital, University of Social Welfare and Rehabilitation Sciences, Tehran, Iran; All India Institute of Medical Sciences - Patna, INDIA

## Abstract

**Background:**

While mental health alterations during active COVID-19 infection have been documented, the prevalence of long-term mental health consequences remains unclear. This study aimed to determine the prevalence of mental health symptoms—depression, anxiety, stress, and suicidal tendencies—and to identify their trends and associated risk factors in individuals with long-COVID.

**Methods:**

We conducted a systematic literature search of databases including PubMed, EMBASE, Scopus, CINAHL, Cochrane Library, Web of Science, and PsycINFO up to August 2024, targeting observational studies published in English. Study quality was assessed using structured standard tools. The primary outcome was the pooled prevalence of depression, anxiety, stress, and suicidal tendencies in individuals with long-COVID. Secondary outcomes included trends in these mental health problems over time and identification of associated determinants.

**Results:**

A total of 94 eligible studies were included in the analysis. The pooled prevalence estimates, regardless of follow up times duration, were as follows: depression, 25% (95%CI:22–28%; PI:1–59%); anxiety (adjusted via trim and fill method), 23%(95%CI:21–25%;PI:2–35%); composite outcomes of depression and/or anxiety, 25% (95%CI:23–27%;PI:2–51%); stress, 26%(95%CI:13–39%;PI:1–69%); and suicidality, 19%(95%CI:15–22%;PI:13–25%). The results of meta-regression analyses revealed a statistically significant trend showing a gradual decrease in the prevalence of the composite outcome of anxiety and/or depression over time (RD = -0.004,P = 0.022). Meta-regression results indicated that being female and younger age were significantly associated with a higher prevalence of mental health symptoms. Study design and study setting did not contribute to heterogeneity.

**Conclusion:**

One-fourth of individual with long-COVID experience mental health symptoms, including depression, anxiety, and stress, which remain prevalent even two years post-infection despite a slight decreasing trend. Factors such as female gender and younger age were linked to higher rates of anxiety and depression. These findings indicate the need for ongoing mental health screening and early interventions to mitigate long-term psychological distress in long-COVID patients.

## Introduction

The COVID-19 pandemic is regarded as an unprecedented natural disaster in the current century, with far-reaching and catastrophic consequences for the global population. As reported by the World Health Organization (WHO), more than 750 million people have been infected globally, and the death toll has tragically surpassed 6 million [1, 2]. While most patients recovered in the weeks following acute infection, evidence quickly emerged that some people reported persistence or appearance of a wide variety of symptoms with variable intensity, regardless of the initial disease severity [1, 3]. The term “long-COVID” was introduced in May 2020 [3]. According to the WHO it was defined as the presence of symptoms lasting for at least 2 months in individuals with a history of probable or confirmed SARS-CoV-2 infection, which usually manifests 3 months from the onset of acute illness, yet cannot be explained by an alternative diagnosis [4].

In this respect, emerging evidence reported a high prevalence of prolonged psychiatric symptoms in individuals who had preliminary COVID-19 or have recovered from the acute infection, that could last for weeks, even months, after recovery [5–7]. For instance, Badenoch et al. (2021) in a meta-analysis reported that among patients experiencing persistent neuropsychiatric symptoms after an initial 2-week infection period (with a median follow-up time of 77 days, ranging from 14 to 182 days), the pooled prevalence of anxiety was 19% [19.1% (13.3–26.8%)] [8].

However, the prevalence of some significant mental health symptoms, such as depression, anxiety, stress, and suicidal tendencies, in individuals with long-COVID, are not fully determined. While some studies have identified specific associated risk factors for mental health problems, a thorough understanding of their prevalence and contributing factors in long COVID patients is still missing. Therefore, the aim of this study was to determine the prevalence and trend of mental health symptoms, including depression, anxiety, stress, and suicidal tendencies, and to identify their associated risk factors among individuals with long-COVID. By addressing this gap, we hope to provide a clearer picture of the mental health issues in long COVID patients and inform better clinical practices and public health strategies.

## Material and methods

### Design and registration

This systematic review and meta-analysis adhere to the 2020 preferred reporting items for systematic reviews and meta-analysis (PRISMA) guidelines [9]. The protocol of this review was registered with the International Prospective Register of Systematic Reviews (PROSPERO): (PROSPERO ID: CRD42022346858).

### Data source, search strategy and study selection

We conducted a comprehensive search across seven electronic databases (PubMed including Medline, EMBASE, Scopus, CINAHL, Cochrane Library, Web of Science, and PsycINFO) from inception to August 2024. Additionally, we manually searched the reference lists of relevant articles, including backward and forward citation searches. All identified studies were exported into EndNote Software for duplicate removal, screening, full-text review, and data extraction. Three independent reviewers conducted title and abstract screening, full-text review was completed by two other independent reviewers against the inclusion and exclusion criteria. Disagreements or conflicts were resolved through discussion and consensus. A detailed information is provided in the S1 File.

### Selection criteria

All observational (cross-sectional, case–control, or cohort) Studies were considered for inclusion in this review if they met the following criteria: (i) reported the occurrence or provided adequate data to estimate the prevalence of outcomes among the general adult population, and (ii) utilized validated measurement tools for outcome assessment. Additionally, studies that recruited college students and healthcare providers were also included. We excluded studies that (i) were non-primary research articles (brief communications, commentary, editorials, and reviews); (ii) did not adhere to the defined criteria for long-COVID; (iii) Did not clearly specify the follow-up duration; (iv) studies published in languages other than English. If studies had overlapping participants and survey periods, then the study with the most detailed and relevant information was used. In cases where studies had overlapping participants and survey periods, preference was given to the study providing the most detailed and relevant information.

### Data extraction

After identifying eligible studies through full-text review, two reviewer (SB-G and RB-Y) extracted data. The third reviewer double-checked 15% subsample of the extracted information to check the consistency. General information that was extracted for each study included: the first author, year of publication, sample size, country, and study population. Information on event occurrences such as severity of preliminary infection, follow up time, measurement tools, and associated factors were also extracted.

### Outcome variables and measures

Long-COVID was defined as conditions that occur in individuals with a history of probable or confirmed SARS-CoV-2 infection 3 months from the onset of COVID-19 that last for 2 months and cannot be explained by an alternative diagnosis [10, 11].

For the endpoint outcomes of the study, including anxiety, depression, stress, and suicide tendency, we utilized the definitions employed in each original study, due to the heterogeneity of definitions used across different studies, at different time points.

### Quality assessment

The quality of the included studies was evaluated using the Newcastle–Ottawa Scale (NOS) for observational studies [12]. The NOS consists of eight items classified into three domains of study population/ group selection, study group comparability of study group, and determination of outcome or exposure. This tool evaluates the methodological quality of studies using a star system, providing a semi-quantitative assessment. Scores range from zero to nine stars for cohort and case-control studies and zero to eight for cross-sectional studies. The studies were categorized into four quality levels based on their scores: high (75–100% scores), moderate (50–74% scores), low (25–49% scores), and very low (0–24% scores). Quality assessment involved two reviewers (CFM and JLK), with a third reviewer (SB-G) double-assessing 15% of the articles to ensure consistency.

### Synthesis of results/data analysis

A narrative synthesis was performed for the results of studies to summarize the determinant associated with outcomes. This involved systematically reviewing and integrating findings from the included studies. Initially, we extracted and tabulated key data from each study, then identified and analyzed recurring themes and patterns across the studies, comparing findings to explore consistencies and discrepancies. Finally, to generate a consistent effect measure across studies, meta-analyses was performed.

Heterogeneity was evaluated using the Chi-square test. Publication bias was also assessed by both funnel plot and Egger’s test. The random effect model was used to estimate the pooled proportion in case of significant heterogeneity. In addition, Freeman-Tukey (double arcsine) transformation for proportions was applied to stabilize the variances. Trim and fill method applied in case of significant publication bias. Meta-regression analysis was also run to assess the effect of extracted determinants as the potential sources of heterogeneity. R software metaphor package version 4.3.3 was used to conduct statistical analysis.

## Results

### Search results and characteristics of the included studies

**Fig 1:**
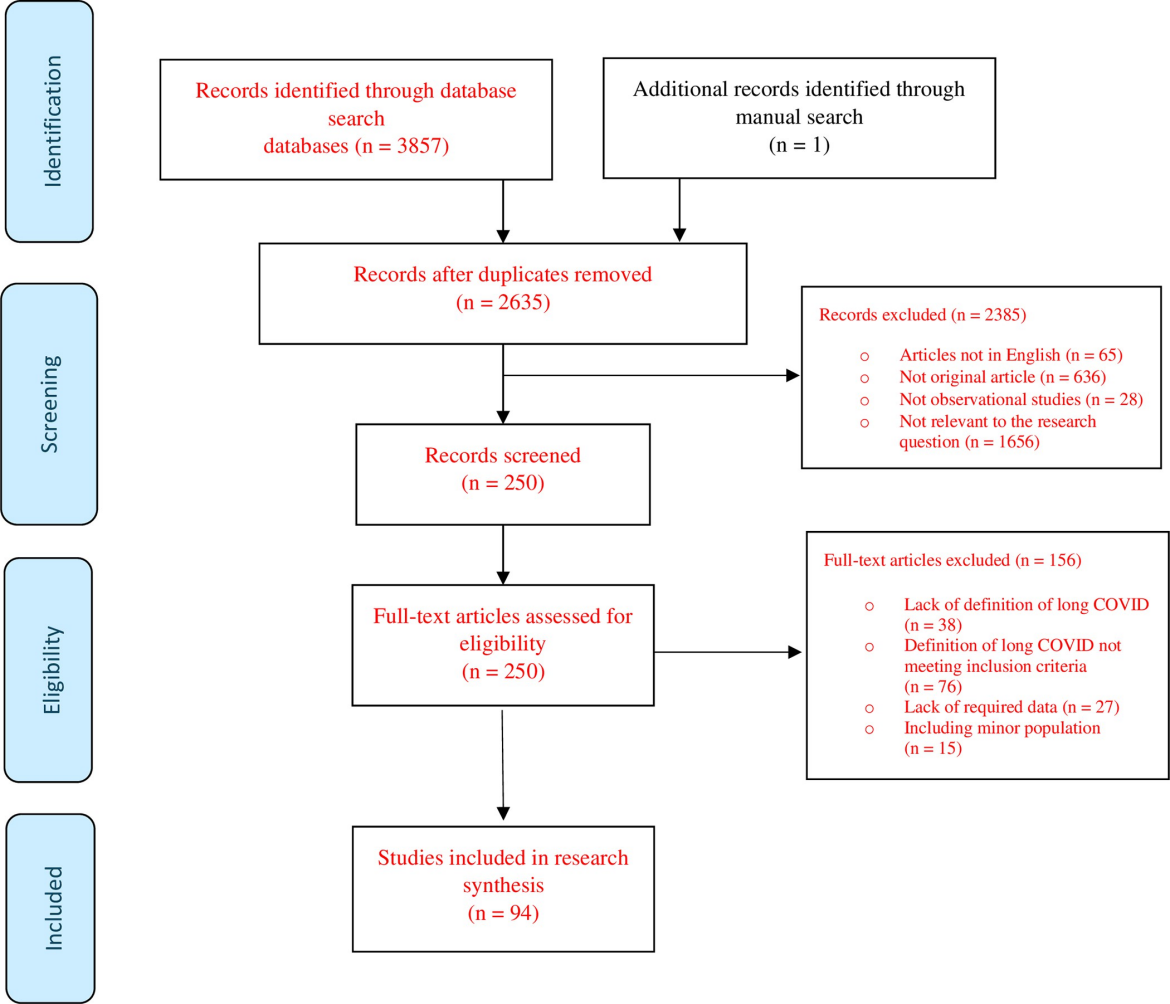
Flowchart of the search process.

The initial literature search yielded 3857 studies, 250 of which were further evaluated by retrieving their full text and 156 of these were excluded. Eventually, 97 eligible studies were included in the systematic review and offered extractable data for the meta-analysis [5–7, 13–103], involving 660484 participants. A flow diagram of this process is present in Fig 1. The main characteristics of included studies are summarized in Table 1.

**Table 1:** Characteristics of studied included.

Author, year	Location	Study design	Study setting	Sample size	Mean age (years)	Male (%)	Day zero	Severity	Follow-up	questionnaire	Outcome	Factors Associated with persistent symptoms *	Factors Associated with Depression or Anxiety or suicide, specifically
PHOSP-COVID Collaborative Group, 2022	UK	cohort study	Multicentre	2320	≥ 18 years, 58.7 (12·5)	64·4	After hospital discharge	ICU	5 months and 1 year	Generalised Anxiety Disorder 7-item scale [GAD-7]), depression (Patient Health Questionnaire-9 [PHQ-9])	Depression, Anxiety	female sex, obesity, invasive mechanical ventilation	-
Abdelrahman MM, 2021	Egypt	cohort study	Single centre	172	17–80 years, 41.8 (17.6)	34.3%	tested positive	Mixed IP/OP/ICU	8–10 month	NM	Depression	Age	-
Abramoff BA, et al. 2023	USA	Observational cohort study	Single centre	324	46.6 (14.0)	31.2%	Date of the first positive COVID test	Mixed IP/OP/ICU	<12 weeks, 12–29 weeks, 30+ weeks	The hospital anxiety scale and Hospital depressions cale	Severe Depression, Severe Anxiety	Poor and African American/ Black individuals	Poor and African American/ Black individuals
Ahmed GK, 2021 Egypt	Egypt	cohort study	Single centre	182	46.49 (17.4)	46.2%	Date of the first positive COVID test	IP/ ICU	6 months	Symptom checklist 90 "SCL 90′′	Depression, Anxiety	being female, diabetes, oxygen support or mechanically ventilated	being female, diabetes, oxygen support or mechanically ventilated
Azizi A, et al. 2022	Morocco	case-control study	Single centre	213	55.1 (16.4)	53.1%	After hospital discharge	IP/ ICU	3 months	The Hospital Anxiety and Depression Scale (HADS)	Anxiety and depression	Older patients, suffering from type 2 diabetes and kidney diseases, admitted to ICU, who stayed a long duration in the hospital, who had severe and longer duration of symptoms and who used Chloroquine	Older patients, suffering from type 2 diabetes and kidney diseases, admitted to ICU, who stayed a long duration in the hospital, who had severe and longer duration of symptoms and who used Chloroquine
Becker C, et al. 2021	Switzerland	prospective cohort study	Single centre (2 hospitals)	90	60 (15.1)	62%	After hospital discharge	IP/ ICU	1 year	The Hospital Anxiety and Depression Scale (HADS)	Depression, Anxiety, Stress	duration of hospitalization, severity of illness, self-perceived overall health status 30 days after hospitalization	-
Bellan M, et al. 2022	Italy	prospective cohort study	Single centre	324	60 (50–69)**	60.5%	After hospital discharge	IP/ ICU	One year	interviewed by an experienced psychiatrist	Depression, Anxiety	diffusing capacity of the lungs for carbon monoxide (DLCO) (> 80%),	-
Brito-Zerón P, et al. 2021	Spain	cohort study	Multi centre	38	54.3 (13.3)	5.3%	Date of the first positive COVID test	Mixed IP/OP/ICU	5 months	NM	Depression and/or Anxiety	raised LDH levels, raised CRP levels, use of hydroxychloroquine and antiviral agents, hospital admission, mean length of hospital admission and requirement of supplemental oxygen	-
Buonsenso D, et al. 2022	Italy	retrospective cohort study	Single centre	155	46.48 (7.3)	49.7%	Date of the first positive COVID test	Mixed IP/OP/ICU	within one year of the acute illness	Ad-Hoc Questionnaire	Anxiety	NM (low quality)	-
Buttery S, et al. 2021	UK	mixed methods approach, both quantitative and qualitative methods	Nationwide	2506	(17 or under to 75 or older)	22%	Date of the first positive COVID test	Mixed IP/OP/ICU	(8–12) weeks and > 12 weeks	NM	Depression and/or Anxiety	NM	-
Cacciatore M, et al. 2022	Italy	secondary analysis of a cohort study	Single centre	83	66.9 (64.2–69.7)**	75.9%	After hospital discharge	IP/ ICU	Within six months after discharge with mean of 3.5 months	The Hospital Anxiety and Depression Scale (HADS)	Depression, Anxiety	NM	-
Cai J, et al. 2023	China	Cohort study	Single centre	21799	43.8 (13.6)	64.47%	After hospital discharge	IP/ ICU	6 months and 12 months	Generalized Anxiety Disorder-7 (GAD-7) questionnaire, Patients Health Questionnaire (PHQ-9)	Depression, Anxiety	Females, youth (age <40 years), middle age (40–60 years), ≥ 2 comorbidities, severe infection in the acute phase	-
Calabria M, et al. 2022	Spain	cross-sectional study	Single centre	136	51.7 (13.5); range (20–88)	36%	Date of the first positive COVID test	Mixed IP/OP/ICU	8 months	Hospital Anxietyand Depression Scale (HADS	Depression, Anxiety	NM	-
Caspersen IH, et al. 2022	Norway	Cohort study	Multi centre	774	NM	42.0%	Date of the first positive COVID test	Mixed IP/OP/ICU	12 months	Ad-Hoc Questionnaire	Depression, Anxiety	NM	-
Catalán IP, et al. 2022	Spain	Cohort study	Single centre	76	(≥18 years)	75%	After hospital discharge	IP/ ICU	12 months	SF‐36 quality of life questionnaire	Depression, Anxiety	NM	-
Clemente I, et al. 2022	Italy	Cohort study	Single centre	48	62.5 (10.1) (range: 40–85)	70.8%	After hospital discharge	IP/ ICU	3 months	Symptom Checklist-90 (SCL90)	Depression, Anxiety	Female	-
Damiano RF, et al. 2023	Brazil	cohort study	single center	710	55 (14.1)	52%	After hospital discharge	IP/ ICU	6–11 month	Hospital Anxiety and Depression Scale (HAD)	Depression	WHO severity, Comorbidity, socio-demographic variables of sex, older age, and lower education level.	Female, lower educational level, Older age
Danesh V, et al. 2023	USA	cohort study	Multi centre	441	51.5	33.1%	Date of the first positive COVID test	Mixed IP/OP/ICU	9 month	A semi-structured interview	Depression, Anxiety	NM	-
de Miranda DAP, et al. 2022	Brazil	longitudinal study	single center	324	42.6% were aged 41–60, 31.5% 21–40, 21.3% 61–80	36 .7%	Date of the first positive COVID test	Mixed IP/OP/ICU	14-months	NM	Depression, Anxiety	Age, severity of SARS-CoV-2 acute infection, presence comorbidities	-
de Oliveira JF, et al. 2022	Brazil	cross-sectional study	single center	369	57 (46–66) **	49%	After hospital discharge	IP/ ICU	2–12 months	EuroQol Group Association five-domain, three-level questionnaire (EQ-5D-3L)	Depression and/or Anxiety	ICU admission, Dysgeusia	-
Delgado-Alonso C, et al. 2022	Spain	Cross-sectional study	single center	50	51.0 (11.6)	26%	Date of the first positive COVID test	Mixed IP/OP/ICU	Minimum 3 months Mean: 9.1 (3.4) months	State-Trait Anxiety Inventory (STAI) and Beck Depression Inventory-II	Depression, Anxiety	NM	-
d’Ettorre G, et al. 2022	Italy	longitudinal cohort study	Single center	137	18–45: 14.6% 46–70: 75.9% 71–85: 9.5%	53.3	After hospital discharge	IP/ ICU	2 years	The EuroQol 5-Dimensions	Depression and/or Anxiety	Female gender, unemployed status, and chronic comorbidities	-
Egger M, et al. 2024	Germany	Observational prospective cohort study	Single center	97	61 (12)	69%	post-discharge	ICU	3, 6, and 12 months	Hospital Anxiety and Depression Scale (HADS),	Depression, Anxiety,	Mechanical Ventilation, Preclinical Frailty, Obesity	Suffering from fatigue, Mechanical Ventilation, Obesity
Fancourt D, et al. 2023	UK	longitudinal study	Multi centre	495	57.3	21.8%	Date of the first positive COVID test	Mixed IP/OP/ICU	over 22 months.	Patient Health Questionnaire (PHQ-9), Generalized Anxiety Disorder assessment (GAD-7)	Depression, Anxiety	NM	-
Fernández-de-Las-Peñas C, 2022	Spain	longitudinal study	Multi centre	1969	61 (16)	53.5%	After hospital discharge	IP/ ICU	mean of 8.4 (1.5) months	Hospital Anxiety and Depression Scale (HADS)	Depression, Anxiety	aged 60–70 years,	-
Fernández-de-Las-Peñas et al. 2024	Spain	Multicenter longitudinal study	Multicenter	1266	61 (16)	54,4%	After hospital discharge	Mixed IP/ICU	6,12 and 18 months	Hospital Anxiety and Depression Scale (HADS-A and HADS-D)	Depression, Anxiety,	No specific risk factors identified	No specific risk factors identified
Ferrando SJ,et al. 2023	USA	Cross-sectional study	single center	75	43.5	29.3%	Date of the first positive COVID test	Mixed IP/OP/ICU	Mean of 220 days	generalized anxiety questionnaire-7 (GAD-7)	Anxiety	NM	-
Frontera JA, et al. 2022	USA	prospective, longitudinal cohort study	Multi centre	451 at 6-month and 383 at 12-month	At 6 months: 69 (57–78)**, at 12 months: 65 (53–73)**	65%	post diagnosis	Mixed IP/OP/ICU	6 and 12-months	Quality of Life in Neurological Disorders (NeuroQoL)	Depression, Anxiety	life stressors including financial insecurity, food insecurity, death of a close contact and new disability, older age, female sex, index COVID-19 severity	life stressors including financial insecurity, food insecurity, death of a close contact and new disability, older age, female sex, index COVID-19 severity
Frontera JA, et al. 2021	USA	prospective, longitudinal cohort study	Multi centre	395	68 (55–77) **	65%	post diagnosis	Mixed IP/OP/ICU	6 months	Quality of Life in Neurological Disorders (Neuro-QoL)	Depression, Anxiety	patients with neurological complications during index hospitalization	patients with neurological complications during index hospitalization
Garout MA, et al. 2022	Saudi Arabia	Cross-sectional study	single center	744	18–29, 138 (18.6%) 30–50, 398 (53.5%) ≥ 50, 207 (27.9%)	49.3%	Date of the first positive COVID test	Mixed IP/OP/ICU	Less than 3 months, 3–6 months, after 6 months	COVID‐19 Yorkshire Rehabilitation Screening (C19‐YRS)	Depression, Anxiety	NM	-
Gasnier M, et al. 2023	France	Cross-sectional study	single center	177	≥18 years, mean; 56 years	41.1%	After hospital discharge	ICU	4 months	Hospital Anxiety and Depression scale-Anxiety subscale (HAD-A), Beck Depression Inventory-13 items (BDI) and Mini International Neuropsychiatric Interview (MINI 5.0)	Depression, Anxiety, suicide risk	Respiratory complaints, Number of long COVID complaints	Respiratory complaints, Number of long COVID complaints
Gil S, et al. 2021	Brazil	cohort study	single center	614	56 (13)	53%	After hospital discharge	IP/ ICU	6 to 11 months	NM	Depression, anxiety	physical inactivity	-
Goldhaber NH, et al. 2022	USA	Cross-sectional study	Multi centre	421	(range: 18–89 years) 52.2 (15)	63.4%	Date of the first positive COVID test	Mixed IP/OP/ICU	331 days (range: 120–617; SD = 84)	Generalized Anxiety Disorder 2-item (GAD-2) and the Patient Health Questionnaire 2-item (PHQ-2)	Depression, anxiety	female sex, COVID-19 hospitalization, poorer pre-COVID self-rated health, younger age	Younger age
Goodman ML, et al. 2023	USA	cross-sectional population survey	Multi centre	621	(range : 20–50 Year) Mean : 36.9	16.5%	Date of the first positive COVID test	Mixed IP/OP/ICU	After 3 month	self-administered questionnaire	suicide ideation, Depression, anxiety	higher daily functional challenges and common mental disorders	-
Gorecka M, et al. 2022	UK	Prospective case–control study	single center	20	45 (13)	47%	Date of the first positive COVID test	Mixed IP/OP/ICU	Afetr 12 weeks	EuroQol Five-Dimensional Five-Level questionnaire (EQ-5D-5L)	Depression and/or Anxiety	NM	-
Gramaglia C, et al. 2022	Italy	cohort study	single center	200	61.5 (51.0–70.5) **	61.2%	After hospital discharge	IP/ ICU	1 year	Beck Depression Inventory, Beck Anxiety Inventory	Depression, anxiety	female gender and depressive symptoms at 4-months follow-up, arterial hypertension, obesity,	female gender and depressive symptoms at 4-months follow-up, arterial hypertension, obesity,
Guo Y, et al. 2023	China	prospective cohort study	Multi centre	208	58 (50.0–64.3) **	48.1%	Date of the first positive COVID test	Mixed IP/OP/ICU	3.3 months, 9.2 months, 18.5 months	Patient Health Questionnaire-9 (PHQ-9)	Depression, anxiety	NM	-
Han JH, et al. 2022	USA	prospective cohort study	Multi centre	213	45 (33, 57) **	34.8%	Date of the first positive COVID test	Mixed IP/OP/ICU	6–11 months	EuroQol visual analogue scale, EuroQol Five-dimensional Five-Level questionnaire Patient Health Questionnaire	Depression and/or Anxiety	poorer long-term health status, poorer quality of life, and psychological distress.	-
Hastie CE, et al. 2022	UK	prospective cohort study	Multi centre	33281	>16 years 45 (31–56) **	39%	Date of the first positive COVID test	Mixed IP/OP/ICU	6, 12 and 18-month	EuroQol-5 Dimension (EQ-5D)	Depression and/or Anxiety	hospitalized infection, ICU admission, age, female sex, deprivation, respiratory disease, and multimorbidity	-
Hellemons ME, et al. 2022	Netherlands	prospective cohort study	single center	92	58.2 (12.3)	63.0%	After hospital discharge	IP/ ICU	3 and 6 months	Hospital Anxiety and Depression Scale (HADS)	Depression, anxiety	NM	-
Herman B, et al. 2022	Indonesia	prospective cohort study	single center	712	NM	40.3%	Date of the first positive COVID test	Mixed IP/OP/ICU	60 days	Patient Health Questionnaire-9 (PHQ-9)	Depression,	Favipiravir prescription, patients living alone	Favipiravir prescription, patients living alone
Holdsworth DA, et al. 2022	UK	prospective cohort study	single center	205	39 (30–46.7) **	84%	Date of the first positive COVID test	Mixed IP/OP/ICU	24 week (IQR17.1–34.0)	Generalized anxiety disorder-7, GAD-7), patient health questionnaire 9	Depression, anxiety	NM	-
Houben-Wilke S, et al. 2022	Netherlands	prospective cohort study	single center	239	50 (39–56) **	18%	Date of the first positive COVID test	Mixed IP/OP/ICU	3 and 6 months	Hospital Anxiety and Depression Scale [HADS]	Depression, anxiety	NM	-
Huang L, et al. 2022	China	longitudinal cohort study	single center	2469	57·0 (48·0–65·0) **	54%	After hospital discharge	IP/ ICU	6, 12 and 24 months	Generalized Anxiety Disorder seven-item scale (GAD-7), the Patient Health Questionnaire 9 (PHQ-9)	Depression and/or Anxiety	NM	-
Huang L, et al. 2021	China	longitudinal cohort study	Single center	1276	59·0 (49–67)**	53	After hospital discharge	IP/ ICU	6 and 12 months	The EuroQol 5-Dimensions five-level (EQ-5D-5L)	Depression and/or Anxiety	Female gender	Female gender
Jiménez-Rodríguez BM, et al. 2022	Spain	longitudinal cohort study	single center	217	59 (49–68) **	53.5%	Date of the first positive COVID test	Mixed IP/OP/ICU	2 and 6 months	NM	Depression	NM	-
Jawad MJ, et al. 2021	IRAQ	cross-sectional study	Multicentre	200	NM	66.5%	tested positive	NM	6 months	Patient Health Questionnaire and State Trait Anxiety Inventory	Depression	male gender,	male gender
Kayaaslan B, et al. 2021	Turkey	longitudinal cohort study	single center	1007	18–34 : (33.1%) 35–49 : (27.0%) ≥ 50 : (39.9%)	54.4%	Date of the first positive COVID test	Mixed IP/OP/ICU	beyond 12 weeks	Ad-hoc questionnaire	Depression, anxiety	Severe acute COVID‐19, hospitalization, and presence of comorbidity	-
Kim Y, et al. 2023	Republic of Korea	prospective online surveys	single center	132	(range: 16–70 years) 38.0 (24.0–50.5) **	31.8%	Date of the first positive COVID test	Mixed IP/OP/ICU	6, 12, and 24 months	EuroQol-5 dimension (EQ5D)	Depression, anxiety	COVID-19 vaccination or the number of vaccinations received may not significantly affect the incidence of long COVID	-
Kim Y, et al. 2022	Republic of Korea	prospective online survey	single center	127	(range: 17–70 years) 37 (26.0–51.0) **	32%	Date of the first positive COVID test	Mixed IP/OP/ICU	6 and 12 months 454 [IQR] 451–458) days	EuroQol-5 dimension (EQ5D)	Depression, anxiety	Older age, female sex, and disease severity	Older age, female sex, and disease severity
Kim Y, et al. 2022	Republic of Korea	prospective cohort study	single center	170	51 (37–61) **	40%	Date of the first positive COVID test	Mixed IP/OP/ICU	6 and 12 months	the Patient Health Questionnaire-9 (PHQ-9), Generalized Anxiety Disorder-7 (GAD-7)	Depression, anxiety	NM	-
Koliadenko N. Vm et al. 2022	Bangladesh	prospective cohort study	single center	129	(range: 20–89) 20–29: 12.4% 30–39: 13.2% 40–49: 25.6% 50–59: 27.9% 60–69: 11.6% 70–79: 7% 80–89: 2.3%	47.2%	Date of the first positive COVID test	Mixed IP/OP/ICU	10 months	Depression, Anxiety, and Stress Scale-21 (DASS-21)	stress, anxiety, depression	NM	-
Kruger A, et al. 2022	South Africa	Cross-sectional study	single center	99	NM	30.3%	Date of the first positive COVID test	Mixed IP/OP/ICU	After 3 months	NM	Depression and/or Anxiety	NM	-
Kucukkarapinar M, et al. 2022	Turkey	prospective cohort study	single center	90	44.58 (15.36)	45.2%	Date of the first positive COVID test	Mixed IP/OP/ICU	After 6 months	Depression, Anxiety and Stress Scale-21 (DASS-21)	stress, anxiety, depression	NM	-
Li D, et al. 2022	China	prospective cohort study	single center	155	43 (34–55) **	52.3%	After hospital discharge	IP/ ICU	up to 2 years	Generalized Anxiety Disorder 7-item (GAD-7) scale, Patient Health Questionnaire-9 (PHQ-9),	anxiety, depression	NM	-
Martino GP, et al. 2022	Italy	prospective cohort study	single center	64	68	64%	After hospital discharge	IP/ ICU	6 and 12 months	NM	anxiety, depression	severe COVID-19	-
Martínez-Cao C, et al. 2021	Spain	prospective cohort study	International	5638	18–29: 8.78% 30–39: 23.04% 40–49: 29.07% 50–59: 24.02% 60–69: 11.56% 70–79: 3.25% 80+: 0.28%	19.97%	Date of the first positive COVID test	Mixed IP/OP/ICU	More than 2 months Median: 190 days (164–229 days)	Patient Health Questionnaire-2 (PHQ-2), Generalized Anxiety Disorder scale-7 (GAD-7)	Depression, Anxiety, suicidality,	younger age, greater reductions in overall health, higher symptom severity, limitations to physical capability, lower income, financial hardship, psychiatric history, employment impact, male sex, men and non-binary gender, and negative experiences with medical professionals, family, friends, partners and employers	younger age, greater reductions in overall health, higher symptom severity, limitations to physical capability, lower income, financial hardship, psychiatric history, employment impact, male sex, men and non-binary gender, and negative experiences with medical professionals, family, friends, partners and employers
Mazza MG, et al. 2021	Italy	prospective cohort study	single center	246	60.1 (12.2)	69%	After hospital discharge	IP/ ICU	6 and 12 months	State-Trait Anxiety Inventory, Zung Severity Rating Scale	anxiety, depression	Gender	Female gender
Mazza MG, et al. 2021	Italy	prospective cohort study	single center	226	(range:26 to 87 y) 58.5 (12.7)	77%	After hospital discharge	IP/ ICU	3 months (90.1 ± 13.4 days)	Zung Self-Rating Depression Scale (ZSDS), 13-item Beck’s Depression Inventory (BDI-13), State-Trait Anxiety Inventory	anxiety, depression	Females, patients with a positive previous psychiatric diagnosis, Duration of hospitalization, presence of psychopathology at one month after discharge	Females, patients with a positive previous psychiatric diagnosis, Duration of hospitalization, presence of psychopathology at one month after discharge
Mendola M, et al. 2022	Italy	prospective cohort study	single center	56	55 (50–61.2) **	50%	After hospital discharge	IP/ ICU	18 months	Ad-hoc questionnaire	Depression and/or Anxiety	NM	-
Menges D, et al. 2021	Switzerland	population-based prospective cohort study	National wide	431	47 (33 to 58) **	50.3%	Date of the first positive COVID test	Mixed IP/OP/ICU	6–8 months Median: 7.2 months (range 5.9–10.3 months)	21-item Depression, Anxiety and Stress Scale (DASS-21)	Depression, anxiety, Stress	NM	-
Morawa E, et al. 2023	Germany	prospective cohort study	single center	110	42.5 (11.9)	31.8%	Date of the first positive COVID test	Mixed IP/OP/ICU	More than 3 months Mean (SD) (13.5 (8.3)	Patient-Health-Questionnaire-9 (PHQ-9)	Depression,	NM	-
Morioka S, et al. 2023	Japan	cross-sectional questionnaire-based survey	Single center	502	48.0 (42.0–55.0) **	40.2	Date of the first positive COVID test	Mixed IP/OP/ICU	6, 12, 18, and 24 months	Ad-hoc questionnaire	Depression,	being female, moderate or severe COVID-19, underlying medical conditions, younger age	younger age
Moy et al. 2022	Malaysia	cross-sectional online questionnaire study	Single center	598	40.2 (10.9)	41.3	Date of the first positive COVID test	Mixed IP/OP/ICU	12 weeks	Patient Health Questionnaire 9 (PHQ-9)	Depression	Female, younger age, being overweight/obese, perceiving to have poorer health	Female, younger age, being overweight/obese, perceiving to have poorer health
Naik H, et al. 2024	USA	Cross-sectional Study	Nationwide	844	46	49.8%	COVID-19 diagnosis date	Mixed IP/OP/ICU	3 months	Patient Health Questionnaire-8 (PHQ-8) and General Anxiety Disorder-7 (GAD-7)	Depression, Anxiety,	NM	NM
Ocsovszky Z, et al. 2022	Hungary	prospective cohort study	single center	166	39.06 (14.49)	43.4	Date of the first positive COVID test	Non-hosptalized OP	mean 25 ± 18 weeks	Beck-Depression Inventory (BDI), Beck Anxiety Inventory (BAI)	anxiety, depression	Depression symptoms during acute infection age, and life, satisfaction, presence of pre-existing affective or anxiety problems	pre-existing mental health problems
O’Kelly B, et al. 2022	Ireland	prospective cohort study	single center	155	43.3 (31–52)	32	Date of the first positive COVID test	Mixed IP/OP/ICU	2–4 and 7–14 months	SF-12 Health Survey (SF-12)	anxiety,	number of initial symptoms	-
Orrù G, et al. 2021	Italy	cross-sectional questionnaire-based survey	Single center	507	<20: 0.20% 20–29: 12.23% 30–39: 20.91% 40–49: 30.77% 50–59: 26.04% 60–69: 8.28% >70: 1.58%	17.9	Date of the first positive COVID test	Mixed IP/OP/ICU	More than 3 months	EuroQol Five-Dimensional Questionnaire: EuroQol-5D (EQ-5D)	anxiety, depression	NM	-
Peter RS, et al. 2022	Germany	Population based, cross sectional study	National wide	12053	(range: 18–65 years) 44.1	41.2 (13.7)	Date of the first positive COVID test	Mixed IP/OP/ICU	6 to 12 months, mean: 8.5 months	SF-12 Health Survey (SF-12)	Depression and/or Anxiety	NM	-
Phu DH, et al. 2023	Thailand	cross-sectional study	Multi center	939	19–59: 84.7% ≥ 60: 15.3%	22.6	Date of the first positive COVID test	Mixed IP/OP/ICU	After 3 months	21-item Depression Anxiety and Stress Scale (DASS-21)	Depression, Anxiety, stress	female patients, medical history, low income,	female patients, medical history, low income,
Qi T, er al. 2021	China	cross-sectional- online survey study	Single center	1171	Male: 31.66 female: 34.61	42.19%	Date of the first positive COVID test	Mixed IP/OP/ICU	more than 1 year	patient health questionnaire-9 (PHQ-9) and generalized anxiety disorder-7 (GAD-7)	Depression, Anxiety,	Living alone, regular exercises (protective), negative attitude towards the pandemic	Living alone, regular exercises (protective), negative attitude towards the pandemic
Richter D, et al. 2022	Germany	cross-sectional- study	Single center	70	50.5 (40–58)**	31.4	Date of the first positive COVID test	OP	more than 3 months	Hospital Anxiety and Depression Scale (HADS)	Depression, Anxiety,	Hypoechogenic brainstem raphe alterations in transcranial sonography (TCS)	Hypoechogenic brainstem raphe alterations in transcranial sonography (TCS)
Román-Montes CM, et al. 2023	Mexico	cross-sectional study	Single center	246	50 (41–63) **	54.87	After hospital discharge	IP/ ICU	After 3 months, 150 days (IQR 90–225)	EuroQol Five-Dimensional Questionnaire: EuroQol-5D (EQ-5D)	Depression and/or Anxiety	Women, tobacco smoking, severity of lung involvement in the initial chest tomography	-
Samper-Pardo M, et al. 2023	Spain	secondary data analysis from a randomized clinical trial	Single center	100	(range: 29–72), 48.2 (9.2)	20%	Date of the first positive COVID test	Mixed IP/OP/ICU	After 3 months, Median of 18 months	Hospital Anxiety and Depression Scale (HADS)	Depression, Anxiety,	Educational level, number of persistent symptoms, affective affectation,	Educational level, number of persistent symptoms, affective affectation
Sayde GE, et al. 2023	USA	prospective cohort study	single center	77	68 (63–73) **	95.2%	After hospital discharge	IP/ ICU	3 and 6 months	9-question Patient Health Questionnaire (PHQ-9), and 7-item Generalized Anxiety Disorder (GAD-7).	Depression, Anxiety,	NM	-
Schandl A, et al. 2021	Sweden	prospective cohort study	single center	113	NM	76.1%	After hospital discharge	ICU	Mean of 5 months	Hospital anxiety and depression scale (HADS)	Depression, Anxiety,	NM	-
Spada MS, et al. 2022	Italy	prospective cohort study	single center	1457	59.4 (13.7)	62.5%	After hospital discharge	Mixed IP/OP/ICU	After 3 months (97.6 ± 48.1 days)	Hospital Anxiety and Depression Scale (HADS)	Depression, Anxiety,	hospitalization–regardless of the setting of care–and promptness in follow-up evaluation had protective effect.	hospitalization–regardless of the setting of care–and promptness in follow-up evaluation had protective effect.
Stallmach A, et al. 2022	Germany	prospective cohort study	single center	355	(range: 17–86), 51 (40, 60) **	40%	Date of the first positive COVID test	Mixed IP/OP/ICU	6 months	Patient Health Questionnaire, PHQ-9	Depression	hospitalization, ICU admission,	hospitalization, ICU admission,
Staudt A, et al. 2022	Germany	prospective cohort study	single center	101	60 (range 28–69)	58%	After hospital discharge	OP/ICU	10 months	Patient Health Questionnaire, PHQ-9	Depression	fatigue, cognitive impairment and low quality of life	fatigue, cognitive impairment and low quality of life
Tabacof L, et al. 2022	USA	cross-sectional study	Single center	156	44 (13–79) **	31%	Date of the first positive COVID test	Mixed IP/OP/ICU	median 351(82–457 days)	generalized anxiety disorder scale (GAD-7), patient health questionnaire-2 (PHQ-2), EuroQol Five-Dimensional Questionnaire: EuroQol-5D (EQ-5D)	Depression, Anxiety, Stress,	NM	-
Talhari C, et al. 2023	Brazil	cross-sectional online survey	Multi center	5791	< 30: 20.8% 31–60: 67.6% > 60: 11.6	25.4	Date of the first positive COVID test	Mixed IP/OP/ICU	12 weeks	Ad-hoc questionnaire	Depression, Anxiety,	Female sex, myalgia, anosmia, and severe disease, Pre-existing depression	Pre-existing depression
Taquet M, 2021	UK	retrospective cohort study	Nationwide	273618	46.3 (19.8)	43.4%	Date of the first positive COVID test	Mixed IP/OP/ICU	6 months	NM	Depression and/or Anxiety	age, sex, or severity of infection	-
Titze-de-Almeida R, et al. 2022	Brazil	prospective cohort study	single center	236	(range: 19–82 y) 41.2 (12.8)	39%	Date of the first positive COVID test	Mixed IP/OP/ICU	5–8 months	Generalized Anxiety Disorder 2-item questionnaire (GAD-2)	Depression, Anxiety,	NM	-
Thanh HN, et al. 2024	Vietnam	Cross-sectional Study	Multicenter	394	29.46 (12.17)	36.3%	NM	Mixed IP/OP/ICU	3 months	Depression, Anxiety, and Stress Scale (DASS-21)	Depression, Anxiety, stress	Female gender and quality of life issues	NM
Tebeka S, et al. 2023	France	Cross-sectional survey	National wide	1095	18–24: 9.8% 25–34: 27.8% 35–44: 19.5% 45–54: 17.7% 55–64: 14.0% ≥65: 11.3%	39%	COVID-19 diagnosis date	Mixed IP/OP/ICU	3 months	Generalized Anxiety Disorder (GAD-2), Patient Health Questionnaire (PHQ-2)	Depression, Anxiety,	Chronic anxiety	Pre-existing chronic anxiety
Tsai J, et al. 2024	USA	Longitudinal cohort study	Multicenter	3595 (511 completed follow-up over 6 months)	38.52	20.41%	day of laboratory-confirmed COVID-19 infection	Mixed IP/OP/ICU	3 months, and 6 months	Patient Health Questionnaire-4	Depression	Being white hispanic ethnicity, older age, Greater Anxiety and Depression Symptoms	Greater Anxiety and Depression Symptoms at beginning
Taquet M, eta l. 2021	UK	retrospective cohort study	National wide	236 379	46 (19·7)	44%	Date of the first positive COVID test	Mixed IP/OP/ICU	6 months	NM	Anxiety	severe COVID-19	severe COVID-19
Veldhuis CB, et al. 2021	USA	prospective cohort study	National wide	1567	18–30: 38.0% 31–40: 31.8% 41–50: 14.2% 51–65: 12.3% 66+: 3.7%	11.8%	Date of the first positive COVID test	Mixed IP/OP/ICU	5 months	Epidemiologic Studies Depression (CES-D), Generalized Anxiety Disorder 7-item (GAD-7) scale	Depression, Anxiety,	Specific demographic groups (people of color and sexual and gender minorities)	Specific demographic groups (people of color and sexual and gender minorities)
Walker S, et al. 2023	UK	cross-sectional study	National wide	3754	47.7 (12.3)	28.2	Date of the first positive COVID test	Mixed IP/OP/ICU	12 weeks or more	Patient Health Questionnaire–Eight Item Depression Scale), (Generalised Anxiety Disorder Scale, Seven-Item	Depression and/or Anxiety	NM	-
Wang Y, et al. 2024	UK	A prospective cohort study using data from the UK Biobank	Nationwide	26101	68.5	44.3%	First positive COVID-19 test	Mixed IP/OP/ICU	12 months	NM	Depression, Anxiety,	Higher risk for those hospitalized, Lower risk for fully vaccinated individuals	Hospitalization
Whiteside DM, et al. 2023	USA	cross-sectional study	Single center	43	48.6 (12.6)	16.3	Date of the first positive COVID test	Mixed IP/OP/ICU	6 months	Personality Assessment Inventory (PAI)	Depression, Anxiety,	NM	-
Wong AW, et al. 2023	Canada	longitudinal cohort study	Multi center	1344	51 (15)	42%	Date of the first positive COVID test	Mixed IP/OP/ICU	3–6 months	The EuroQol 5-Dimensions (EQ5D)	Depression,	NM	-
Zhao Y, et al. 2021 ^1003^	China	prospective cohort study	Multicenter	94	48.11	58%	After hospital discharge	IP/ ICU	One year	24 items, Hamilton Depression Rating Scale (HAMD-24)	Depression, Anxiety,	Age, Pulmonary structural abnormalities and pulmonary diffusion capacities	-
Zhang L, et al. 2022	China	Cross-sectional Study	Single center	255	43.78 (16.0)	50.9%	After hospital discharge	IP/ ICU	1 year	The EuroQol 5-Dimensions (EQ5D)	Depression and/or Anxiety	Older age, Number of post-COVID-19 symptoms	Older age, Number of post-COVID-19 symptoms

* It could include depression, anxiety, or suicidality

IP, inpatient;OP,outpatient;ICU,intensivecareunit;EMR,electronicmedicalrecords.

**IQR

Fifty studies were conducted in Europe including Norway, UK, Switzerland, Italy, Spain, Germany, Hungary, Turkey, Netherlands, France, Ireland, Sweden [5, 6, 13, 19–22, 24–27, 32–34, 39, 43, 44, 47–50, 52, 53, 58, 60–70, 73, 75, 77, 78, 80, 81, 84, 87, 91, 92, 96–99, 102], fifteen in the United States and Canada [16, 29, 35–37, 41, 42, 46, 76, 82, 86, 88, 89, 101, 103], nineteen in Asia including Iraq, Thailand, China, Malaysia, Japan, Bangladesh, Republic of Korea, Indonesia, Saudi Arabia and Vietnam [7, 14, 23, 38, 45, 51, 54–56, 59, 66, 71, 72, 90, 93–95, 100, 104], four in Africa including Egypt, Morrocco, south Africa [15, 17, 18, 57], and six in South America including Brazil, Mexico [28, 30, 31, 40, 74, 83]. Follow-up time ranged from 2 to 24 months (Table 1).

All included studies reported on the prevalence of depression or anxiety, with only seven studies also addressing stress [5, 58, 64, 71, 82, 100, 104] and two studies examining the prevalence of suicidality [39, 42].

### Quality appraisal, publication bias and heterogeneity

The quality appraisal of the included studies is presented in S1-S3 Tables in S1 File. Among them, 32.9% (31/94) of studies were judged as high quality, 56.3% (53/94) were assessed as moderate quality, and 10.6% (10/94) were classified as low quality. None of the studies were considered very low quality, resulting in all studies being included in the final analysis.

**Fig 2:**
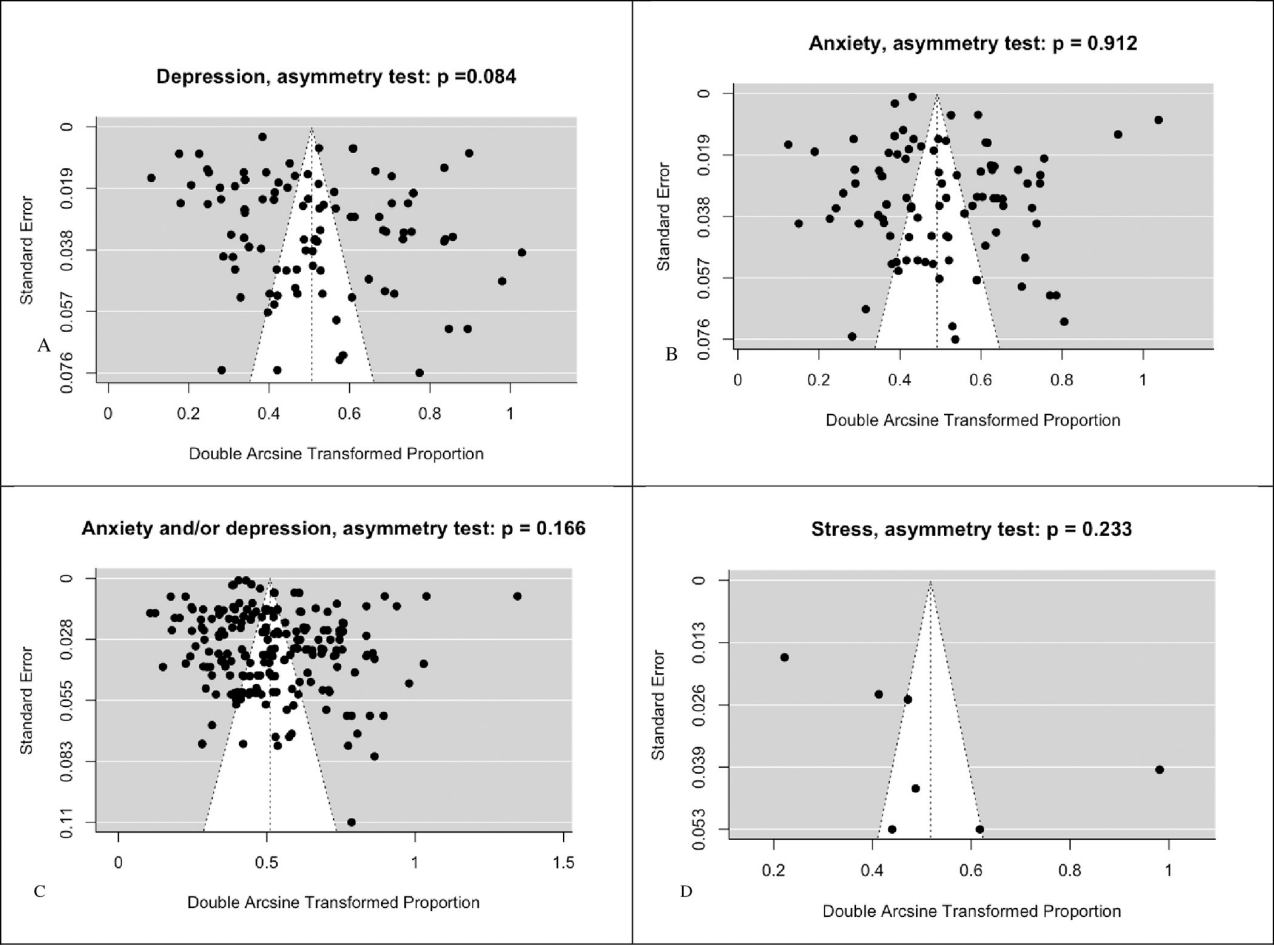
Funnel plot for testing asymmetry hypothesis using Freeman-Tukey double arcsine transformation for proportions. A: Depression, B: Anxiety, C: Anxiety and/or Depression D: Stress.

Based on the funnel plot (Fig 2) and Egger’s test (Table 2), no statistically significant publication bias was observed among the studies for any of the outcomes. Due to insufficient data, publication bias for the outcome of suicidality was not performed. Heterogeneity tests for all outcomes were statistically significant (P < 0.001); therefore, all estimations were obtained using the random effects method.

**Table 2:** Results of random effect meta-analysis using Freeman-Tukey double arcsine transformation for proportions, regardless of the duration of their symptoms.

Outcome	Heterogeneity Test* P-value	Publication bias test** P-value	Pooled Pevalence^€^ (95%CI) (95%PI)
Depression	<0.001	0.084	0.25
			CI: (0.22, 0.28)
			PI: (0.01, 0.59)
Anxiety	<0.001	0.912	0.23
			CI: (0.21, 0.25)
			PI: (0.02, 0.35)
Composite outcome of Anxiety and/or depression	<0.001	0.167	0.25
			CI: (0.23, 0.27)
			PI: (0.02, 0.51)
Stress	<0.001	0.233	0.26
			CI: (0.13, 0.39)
			PI: (0.01, 0.69)
Suicidality	<0.001	^$^--	0.19
			CI: (0.15, 0.22)
			PI: (0.13, 0.25)

The significance level was set at α = 0.05.

CI: Confidence Interval; PI: Prediction Interval.

^€^ obtained from inverse-variance weighting approach with the Freeman–Tukey double arcsine transformation

* Obtained from Chi squared test

** Egger test

^$^ Insufficient data

### Results of meta-analysis, subgroup analysis and meta-regression analysis

Table 2 provides a summary of the overall prevalence of depression, anxiety, stress, and suicidality in individuals with long-COVID, regardless of the duration of their symptoms depression, 25% (95% CI: 22–28%; PI:1–59%); anxiety (adjusted via trim and fill method), 23% (95% CI:21–25%; PI: 2–35%); composite outcomes of depression and/or anxiety, 25% (95% CI: 23–27%; PI: 2–51%); stress, 26% (95% CI: 13–39%; PI: 1–69%); and suicidality, 19% (95% CI: 15–22%; PI: 13–25%).

**Fig 3:**
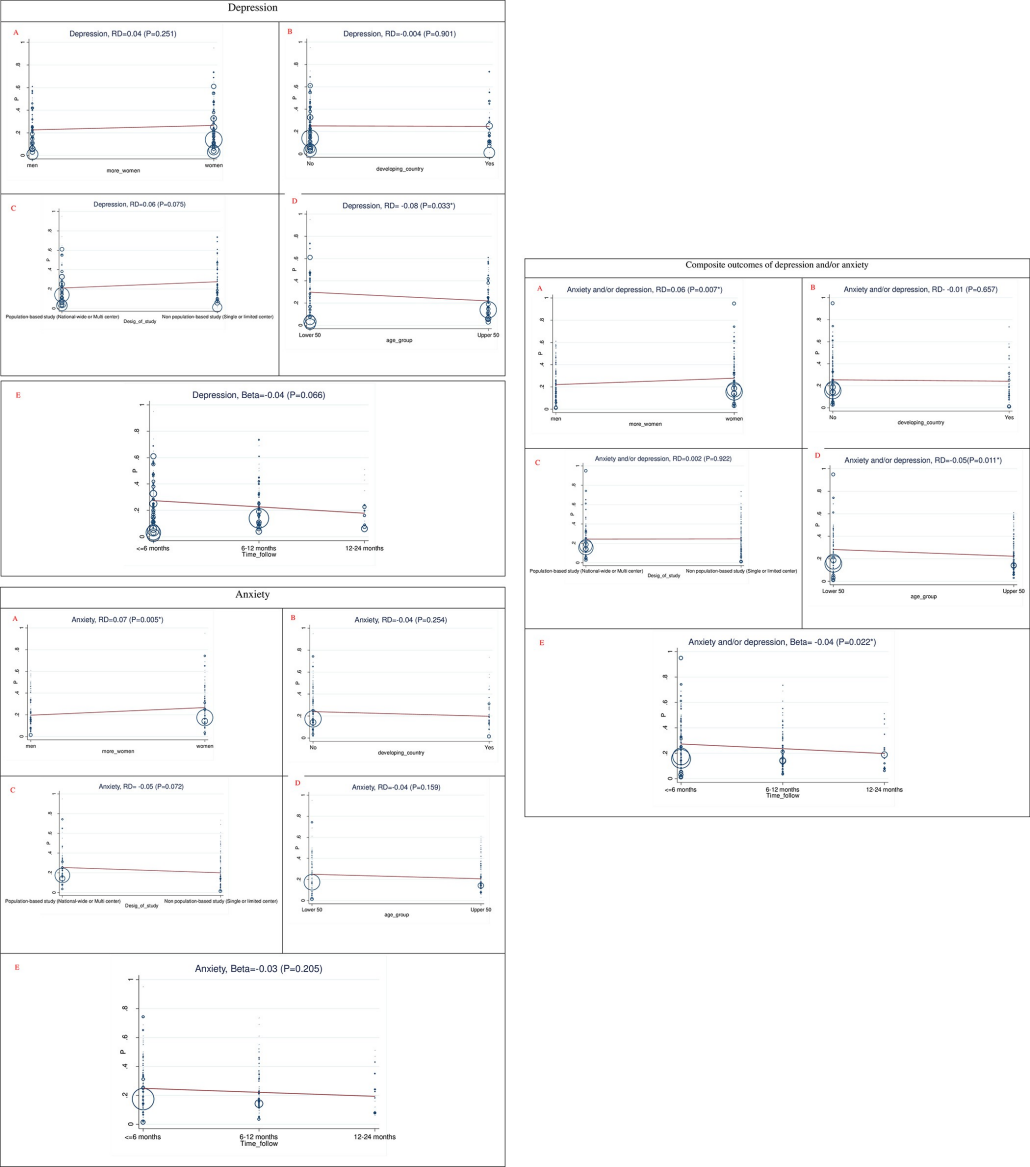
Subgroup and meta-regression analysis of depression, anxiety, and composite outcomes of depression and/or anxiety results stratified by: (A) gender (Studies with more women participants vs, Studies with more men participants), (B) Study setting (living in developing vs. developed countries), (C) Study design, (population-based vs. non-population-based), (D) Age (< = 50 yr vs. >50 yr), and (E) Follow-up time (up to 6 months, 6–12 months, and 12–24 months) in individuals with long covid. RD: risk difference.

The results of the subgroup and meta-regression analyses, stratified by follow-up duration, age, gender, study design, and study setting, are summarized in Table 3 and Fig 3. Regarding the depression, the pooled prevalence of depression at follow-up intervals of up to 6 months, 6–12 months, and 12–24 months was 27% (95% CI: 23–31%), 23% (95% CI: 20–26%), and 15% (95% CI: 9–21%), respectively, exhibiting a non-significant decreasing trend over time (Beta = -0.04, P = 0.06). Meta-regression analyses revealed studies with a higher proportion of female participants reported a significantly higher prevalence of depression compared to those with a greater proportion of male participants (27% vs. 20%, P = 0.025). Additionally, individuals aged ≤50 years exhibited a significantly higher prevalence of depression compared to those >50 years (27% vs. 22%, P = 0.033). Population-based studies reported a slightly lower prevalence compared to non-population-based studies (21% vs. 28%, P = 0.07), though this difference was not statistically significant. Finally, there were minimal setting-related differences, with a pooled prevalence of 25% for studies conducted in both developing and non-developing countries, showing no significant differences by study setting.

**Table 3:** The results of the subgroup and meta-regression analyses, stratified by follow-up duration, age, gender, study design, and study setting.

		Pooled Prevalence (95% CI)		
Subgroups		Depression	Anxiety	Composite outcome of Anxiety and/or depression
Follow up time	up to 6 ^months^	0.27 (0.23, 0.31)	0.25 (0.21, 0.29)	0.27 (0.25, 0.30) *
	6–12 ^months^	0.23 (0.20, 0.26)	0.21 (0.19, 0.23)	0.23 (0.21, 0.24)
	12–24 ^months^	0.15 (0.09, 0.21)	0.21 (0.12, 0.29)	0.20 (0.16, 0.24)
Gender	Studies with more women participants	0.27 (0.23, 0.30)	0.27 (0.23, 0.30) *	0.28 (0.25, 0.30) *
	Studies with more men participants	0.22 (0.19, 0.26)	0.20 (0.17, 0.22)	0.22 (0.20, 0.24)
Age^€^	< = 50 yr	0.27 (0.22, 0.31) *	0.25 (0.19, 0.31)	0.28 (0.25, 0.32) *
	>50 yr	0.22 (0.20, 0.23)	0.20 (0.18, 0.22)	0.22 (0.20, 0.23)
Study design	Population- based design	0.21 (0.17, 0.25)	0.25 (0.21, 0.29)	0.24 (0.21, 0.27)
	Non Population- based design	0.28 (0.23, 0.32)	0.20 (0.17, 0.22)	0.24 (0.22, 0.27)
Study setting	Developing country	0.25 (0.19, 0.31)	0.20 (0.13, 0.27)	0.24 (0.20, 0.28)
	Developed country	0.25 (0.22, 0.28)	0.24 (0.21, 0.26)	0.25 (0.23, 0.27)

* Significant results

^€^ Mean age of participants in included studies

Regarding anxiety, the pooled prevalence at follow-up duration of up to 6 months, 6–12 months, and 12–24 months was 25% (95% CI: 21–29%), 21% (95% CI: 19–23%), and 21% (95% CI: 12–29%), respectively, showing a non-significant decreasing trend over time (Beta = -0.03, P = 0.205). Meta-regression analyses revealed that studies with a higher proportion of female participants reported a significantly higher prevalence of anxiety compared to those with a higher proportion of male participants (27% vs. 20%, P = 0.025). While individuals aged ≤50 years had a non-significantly higher prevalence of anxiety compared to those >50 years (25% vs. 20%, P = 0.159), this difference did not reach statistical significance. Population-based studies reported a slightly lower prevalence compared to non-population-based studies (25% vs. 20%, P = 0.072), although this difference was also not statistically significant. Lastly, studies conducted in developed countries had a higher, but non-significant, pooled prevalence of anxiety compared to those in developing countries (24% vs. 20%, P = 0.254).

The composite outcome of depression and/or anxiety demonstrated a consistent pattern, with pooled prevalence rates at follow-up intervals of up to 6 months, 6–12 months, and 12–24 months being 27% (95% CI: 25–30%), 23% (95% CI: 21–24%), and 20% (95% CI: 16–24%), respectively, indicating a significant decreasing trend over time (Beta = -0.04, P = 0.022). Studies with a higher proportion of female participants reported a significantly higher prevalence of the composite outcome compared to those with a higher proportion of male participants (28% vs. 22%, P = 0.007). Meta-regression analyses revealed that individuals aged ≤50 years exhibited a significantly higher prevalence compared to those >50 years (28% vs. 22%, P = 0.011). Setting-related and study design differences were minimal, with both population-based and non-population-based studies showing a pooled prevalence of 24%, and studies from developing and developed countries showing pooled prevalences of 24% and 25%, respectively, with no statistically significant difference.

Due to a lack of data, we could not perform those analysis for stress and suicidality.

### Determinants of outcomes

Moste of the studies presented factors associated with persistent psychological symptoms in patients with long-COVID which categories as 4 factors, including:

#### (i) Demographic and socioeconomic factors

It was included: male gender [14, 96], female gender [17, 28, 36, 44, 55, 61, 62, 71, 93, 94], Older patients [18, 28, 36, 55, 90], younger patients [41, 66, 94, 96], lower educational level [28, 75], life stressors including death of a close contact and new disability, negative experiences with medical professionals, family, friends, partners and employers [36, 96], patients living alone [7, 72], Specific demographic groups (people of color such as African American/ Black individuals and sexual and gender minorities such as non-binary gender) [16, 83, 96];

#### (ii) Lifestyle and health status factors

It was included: lower income and financial insecurity [16, 71, 96], unemployment [96], inactivity [72, 96], low quality of life [81], Suffering from one or multi co-morbidities [71] such as type 2 diabetes [17, 18], kidney diseases [18], Respiratory complaints [39], arterial hypertension, obesity [44, 94, 99], previous psychiatric diagnosis or pre-existing depression or anxiety [62, 67, 83, 96, 97] and negative attitude towards the pandemic [72], perceiving to have poorer health [94, 96];

#### (iii) Acute COVID-19 infection characteristics

It was included: oxygen support or mechanically ventilated [17, 99], admitted to Intensive Care Unit (ICU) [18, 80], hospitalization or stayed a long duration in the hospital [18, 80, 102], severe and longer duration of symptoms in acute phase [18, 36, 55, 62, 91, 96, 103], used Chloroquine [18], neurological complications during index hospitalization [36], Favipiravir prescription [7], Outpatient care [78];

#### (iv) Disease progression factors

It was included: numbers of persistent symptoms [39, 75, 90], presence of psychopathology such as depressive symptoms, fatigue and cognitive impairment after acute phase [44, 62, 81, 99], Hypoechogenic brainstem raphe alterations in transcranial sonography (TCS) [73], affective affectation [75].

## Discussion

This systematic review and meta-analysis estimated the prevalence of significant mental health symptoms in individuals with long-COVID. Additionally, it provided evidence on the trends of these problems over time and identified key determinants. The results of our study showed that one-fourth of patients suffering from long-COVID experienced mental health symptoms, including depression, anxiety, and stress. Additionally, although the results were inconclusive due to a lack of data, one-fifth of these patients exhibited suicidality. Furthermore, longer follow-up, up to 24 months, indicated that the trend of these symptoms slightly decreased over time. Being a woman and younger age were significantly associated with a higher prevalence of mental health issues.

After a while since the first cases emerged, the direct effects of COVID-19 on mental health were starting to become apparent [105]. Uncertainty, social distancing, economic disruptions, stringent infection control, and national lockdowns and disruption to mental health services induced by the COVID-19 pandemic, led to heightened levels psychological challenges [106, 107]. In one of the preliminary reports, Nochaiwong et al. (2020) conducted a systematic review and meta-analysis of 107 observational studies spanning 32 countries. Their findings revealed prevalence estimates of 28.0% for depression, 26.9% for anxiety, and 36.5% for stress among individuals who had experienced preliminary COVID- 19 infection. These results highlighted a notable increase in the prevalence of common mental health problems during the pandemic compared to the pre-COVID-19 era [108].

Nevertheless, emerging evidence has demonstrated that a spectrum of sequelae and complications can persist long after the acute infection, irrespective of the severity of the initial illness, collectively referred to as long- COVID [109]. Similar to acute COVID-19, long- COVID can affect multiple organs and systems, resulting in a wide range of symptoms, including mental health symptoms.

While accurately reporting long-COVID symptoms is challenging due to accuracy of diagnosis, reporting systems, population assessed, accuracy of self-reporting and length of follow-up period, our meta-analysis revealed that approximately 25% of patients with persistent symptoms experience mental health problems including anxiety and/or depression and stress. This condition may pose a significant public health concern, affecting millions of individuals worldwide. In a recent published study, Zheng et al. (2023), in a meta-analysis and systematic review of 40 studies with 12,424 individuals, reported that the pooled prevalence of any long COVID and psychiatric symptoms in children and adolescents with long covid were 23.36% (95% CI: 15.27–32.53) and (12.30%, 95% CI: 5.38–21.37), respectively [110].

Although the underlying mechanisms of mental problems in individual with COVID remain unclear, some evidence suggests that the virus can infect the central nervous system (CNS) via hematogenous or neuronal retrograde neuro-invasive routes. This infection can also induce septic encephalopathy and may affect the permeability of the blood-brain barrier, allowing peripheral cytokines and other blood-derived substances to enter the CNS and contribute to neuroinflammation [111–113].

Additionally, the pandemic itself has had a negative impact on mental health. Living through a global health crisis, experiencing hospitalization, quarantine, isolation, social distancing, loneliness, reduced physical activity, and financial insecurity all potentially increase the risk of anxiety and depression [107, 111]. These processes may collectively contribute to the development of long-term mental health issues following COVID-19.

Despite inconclusive results due to data limitations, our study found a concerning prevalence of suicidality among individuals with long-COVID, affecting one-fifth of the patient population. Suicidality is a complex outcome influenced by multiple factors, including the interplay of physical health sequelae, psychological distress, and social determinants [114, 115]. Future longitudinal studies with robust methodologies are warranted to confirm this finding and to elucidate the causal pathways and identify effective strategies for suicide prevention in this vulnerable population.

Furthermore, although we observed a gradual decrease in the prevalence of mental health problems over time, the prevalence remained high even after 24 months of follow-up. This persistent prevalence underscores the long-term impact of COVID-19 on mental health and highlights the need for sustained mental health support for these individuals. However, the slight yet significant decrease in mental health symptoms may reflect natural recovery processes or the positive effects of ongoing medical and psychological interventions. However, the high prevalence of these issues after two years suggests that many individuals continue to experience substantial psychological distress long after their initial COVID-19 infection.

In addition, our systematic review identified several key factors associated with persistent psychological symptoms in individuals with Long COVID. These factors can be broadly categorized into four main domains of demographic and socioeconomic factors, lifestyle and health status factors, acute COVID-19 infection characteristics, and disease progression factors. These findings highlight the multifaceted nature of psychological sequelae in Long COVID, influenced by a complex interplay of demographic, socioeconomic, health-related, and disease-specific factors, which is crucial for informing clinical practice and public health strategies. Specifically, being female and residing in developed countries were significantly linked to increased rates of depression among individuals with long-COVID. This gender disparity may reflect broader trends observed in mental health, where females are generally more susceptible to depression due to a combination of biological, hormonal, and psychosocial factors. The higher prevalence of depression in developed countries could be attributed to various factors. These include greater awareness and reporting of mental health issues, better diagnostic capabilities, and possibly higher levels of stress and lifestyle changes associated with the pandemic in these regions.

We observed a lower prevalence of these mental health symptoms in population-based studies compared to clinical or hospital-based studies. This difference likely reflects the broader inclusion of asymptomatic and mild cases in population-based research, whereas clinical studies tend to focus on more severe COVID-19 cases prone to persistent mental health issues.

In the current meta-analysis, we assessed publication bias across the included studies. Except for anxiety, where some evidence of publication bias was detected, no significant publication bias was found for depression, stress, or suicidality. For anxiety, the bias was adjusted using the trim and fill method, ensuring that the overall findings remain robust and reliable.

It should be noted that the included studies exhibited heterogeneity. Subgroup analysis suggested that variations in populations, particularly younger age, follow-up duration and female gender, may contribute to this heterogeneity. However, other potential factors, such as ethnicity, socioeconomic status, and lifestyle, could also play a role but were not fully explored. Additionally, the use of different questionnaires and assessment tools to gather data likely contributed to the observed heterogenicity. Variations in study design, sample size, and regional healthcare practices might also have played a role in increasing heterogeneity among the studies.

Our study had some limitations. Despite conducting an extensive literature search, certain unpublished studies that and those written in languages other than English were not included. Additionally, the number of studies that assessed stress and suicidality prevalence was limited, restricting our ability to perform additional analyses. Furthermore, different tools have been used to assess mental health symptoms across studies, leading to potential inconsistencies and variations in reported prevalence rates. Additionally, data on individuals who had severe forms of COVID-19 and subsequently died are lacking. This absence potentially underrepresents the most severe cases in existing research. Furthermore, there were limitations in the data collected, as most of the papers did not present data related to determinants and associated factors specifically for mental health in individuals with long-COVID. Finally, the findings do not generalize to patients who have had COVID-19 but were not diagnosed.

## Conclusion

In conclusion, we found that one-fourth of individuals with long-COVID suffer from mental health symptoms, including depression, anxiety, and stress. Although these symptoms showed a slight decreasing trend over time, their prevalence remained high even two years after the initial infection. While various factors contribute to mental health symptoms in these patients, our analysis highlighted that women and being younger are particularly vulnerable to higher rates of anxiety and depression. These findings emphasize the need for screening and providing adequate support or early intervention for individuals with long COVID to address and reduce long-term psychological distress.

## Supporting information

S1 Checklist PRISMA 2020 checklist. (DOCX)

S1 File Supplementary material. (DOCX)

S2 File Excluding table. Final.
(DOC)
