# Supplementary material for: Prevalence of depression, anxiety, stress, and suicide tendency among individual with long-COVID and determinants: A systematic review and meta-analysis
Source: PLoS One. 2025 Jan 28;20(1):e0312351. doi: 10.1371/journal.pone.0312351 (PMC11774403; doi:10.1371/journal.pone.0312351)
Supplement: S1 File — (DOCX) [file pone.0312351.s002.docx]

**Supplementary material**

**Search strategy**

We performed a comprehensive search across seven electronic databases (PubMed including Medline, EMBASE, Scopus, CINAHL, Cochrane Library, Web of Science, and PsycINFO) from their inception until August 2023. The search strategy was designed to identify observational studies that examined the prevalence of mental health symptoms in individuals with long-COVID. The detailed search terms and keywords used were as follows:

The following search terms/keywords were used to identify relevant studies and titles, abstracts and/or keywords were searched: (“mental health” OR “mental disorder*” OR “mental illness” OR “psychiatr*” OR “psychological health” OR “psychological distress” OR “psychological impact” OR “psychological outcomes” OR “psychological consequence*” OR “psychological comorbid*” OR “psychosocial problem*” OR psychological disorder* OR mood disorder OR depress* OR MDD OR “major depress*” OR suicide OR suicid* OR “attempted suicide” OR “deliberate self-harm” OR “self injurious behavior” OR "self mutilation" OR "self injur*" OR "self mutil*" OR "self poison*" OR “suicidal ideation” OR “death wish” OR “passive suicidal ideation” OR “suicidal behavior” OR “suicide attempt” OR automutilation OR self-poisoning OR Stress” OR Anxiety OR anxious OR stress OR psychological stress*) AND (“long COVID” OR “long COVID-19” OR “long-COVID” OR “long- COVID-19” OR “long haul” OR “long hauler” OR “long haulers” OR “long-haul” OR “long-hauler” OR “long-haulers” OR “chronic COVID” OR “chronic covid-19” OR “post-acute COVID” OR “post-acute covid-19” OR “post acute COVID” OR “post acute covid-19” OR “persistent COVID” OR “persistent covid-19” OR “post-COVID” OR “post-covid-19” OR “post COVID” OR “post covid-19” OR “sequela” OR “sequelae” OR “long-term” OR “long term” OR “COVID syndrome” OR “COVID-19 syndrome” OR “post-acute COVID-19 syndrome” OR “persistent symptom” OR “persistent symptoms” OR “PASC” OR “PACS” OR “PPCS” OR “post-acute” OR “post acute” OR “Survivor”). The search string was tailored and adapted to each database to ensure optimal retrieval of relevant articles.

Supplementary Table 1. Quality assessment of the included studies using the Newcastle–Ottawa Quality Assessment Scale for cohort studies.

|  | **SELECTION** | | | | **COMPARABILITY** | **Outcome** | | | **Total scores** |
| --- | --- | --- | --- | --- | --- | --- | --- | --- | --- |
| **Author, Year** | Representativeness of the exposed cohort  a) Truly representative *  b) Somewhat representative *  c) Selected group  d) No description of the derivation of the cohort | Selection of the non-exposed cohort  a) Drawn from the same community as the exposed cohort *  b) Drawn from a different source  c) No description of the derivation of the non exposed cohort | Ascertainment of exposure  a) Secure record (e.g., surgical record) *  b) Structured interview *  c) Written self report  d) No description  e) Other | Demonstration that outcome of interest was not present at start of study  a) Yes *  b) No | Comparability of cohorts on the basis of the design or analysis controlled for confounders  a) The study controls for age, and BMI *  b) Study controls for other factors (list) *  c) Cohorts are not comparable on the basis of the design or analysis controlled for confounders | Assessment of outcome  a) Independent blind assessment) *  b) Record linkage *  c) Self report  d) No description  e) Other | 2) Was follow-up long enough for outcomes to occur  a) Yes *  b) No | Adequacy of follow-up of cohorts  a) Complete follow up- all subject accounted for *  b) Subjects lost to follow up unlikely to introduce bias- number lost less than or equal to 20% or description of those lost suggested no different from those followed. *  c) Follow up rate less than 80% and no description of those lost  d) No statement |  |
| PHOSP-COVID Colloborative group, 2022 | * | * | * | * | *  * | * | * | * | 9 |
| Calabria, Garcia-Sanches, Grunden et al., 2022 |  |  | * | * | * | * | * | * | 6 |
| Zhao, Yang, An et al., 2021 |  |  | * | * | ** | * | * | * | 7 |
| Re’em, Stelson, Davis et al., | * | * | * | * | ** | * | * |  | 8 |
| Capersen, Magnus, Trogstad, 2022 | * | * | * | * | ** | * | * | * | 9 |
| Catalan, Marti, Sota et al., 2021 |  |  | * | * | ** | * | * | * | 7 |
| Clemente, Sinatti, Cirella et al., 2022 |  |  | * | * | ** | * | * | * | 7 |
| Damiano, Rocca, Serafim et al., 2023 |  |  | * | * | ** | * | * |  | 6 |
| Danesh, Arroliga, Bourgoies et al., 2022 | * | * | * | * | ** | * | * | * | 9 |
| Egger et al., 2024 | * |  | * | * | ** | * | * | * | 8 |
| Miranda, Gomes et al., 2022 |  |  | * | * | ** | * | * | * | 7 |
| Fancourt et al., 20 | * | * | * | * | ** | * | * | * | 9 |
| Fernandez-de-las- Penas et al., 2024 | * |  | * | * | ** | * | * | * | 8 |
| Jawad et al., 2021 |  |  | * | * | * | * | * | * | 6 |
| Fernandec-de-la-Penas et al., 2022 | * | * | * | * | ** | * | * | * | 9 |
| Frontera et al., 2022 | * | * | * | * | ** | * | * |  | 8 |
| Frontera et al., 2021 | * | * | * | * | ** | * | * |  | 8 |
| Garout et al., 2022 |  |  | * | * | ** | * | * | * | 7 |
| Gil et al., 2023 |  |  | * | * | ** | * | * | * | 7 |
| Gramaglia et al., 2022 |  |  | * | * | * | * | * |  | 5 |
| Guo et al., 2023 | * | * | * | * | ** | * | * |  | 8 |
| Han et al., 2022 | * | * | * | * | ** | * | * |  | 8 |
| Hastie et al., 2022 | * | * | * | * | ** | * | * |  | 8 |
| Hellemons et al., 2021 |  |  | * | * | ** | * | * | * | 7 |
| Herman et al., 2022 | * | * | * | * | ** | * | * | * | 9 |
| Holdsworth et al., 2022 |  |  | * | * | ** | * | * | * | 7 |
| Hoben-Vilke et al., 2022 |  |  | * | * | ** | * | * | * | 7 |
| Huang et al., 2022 |  |  | * | * | ** | * | * |  | 6 |
| Jimenez-Rodrigoues et al., 2022 |  |  | * | * | ** | * | * |  | 6 |
| Kayaaslan et al., 2021 |  |  | * | * | ** | * | * | * | 7 |
| Kim et al., 2023 |  |  | * | * | ** | * | * |  | 6 |
| Kim et al., 2022 |  |  | * | * | ** | * | * |  | 6 |
| Kim et al., 2022 |  |  | * | * | ** | * | * | * | 7 |
| Koliadenko et al., 2022 |  |  | * | * | ** | * | * | * | 7 |
| Kruger et al., 2022 |  |  | * | * | ** | * | * | * | 7 |
| Kucukkarapinar et al., 2022 |  |  | * | * | ** | * | * | * | 7 |
| Li et al., 2022 |  |  | * | * | ** | * | * | * | 7 |
| Martino et al., 2023 |  |  | * | * | ** | * | * |  | 6 |
| Mazza et al., 2022 |  |  | * | * | ** | * | * |  | 6 |
| Gennaro et al., 2021 |  |  | * | * | ** | * | * |  | 6 |
| Mendola et al., 2022 |  |  | * | * | ** | * | * | * | 7 |
| Menges et al., 2021 | * | * | * | * | ** | * | * | * | 9 |
| Ocsovszky et al., 2022 |  |  | * | * | ** | * | * | * | 7 |
| O’Kelly et al., 2022 |  |  | * | * | ** | * |  |  | 5 |
| Orru et al., 2021 | * | * | * | * | ** | * |  |  | 7 |
| Abdelrahman et al, 2021 |  |  | * | * | ** |  | * |  | 5 |
| Samper-Pardo et al, 2023 |  |  | * | * | ** | * | * |  | 6 |
| Sayde et al, 2023 |  |  | * | * | ** | * | * |  | 6 |
| Schandl et al, 2021 |  |  | * | * | ** | * | * |  | 6 |
| Spada et al, 2022 |  |  | * | * | ** | * | * |  | 6 |
| Stallmach et al, 2022 |  |  | * | * | ** | * | * | * | 7 |
| Talhari et al, 2023 | * | * | * | * | ** | * | * | * | 9 |
| Taquet et al, 2021 | * | * | * | * | ** | * | * | * | 9 |
| Becker et al, 2021 |  |  | * | * | ** | * | * | * | 7 |
| Titze-de-Almeida et al, 2022 |  |  | * | * | ** | * | * | * | 7 |
| Tsai J, et al. 2024 | * | * | * | * | ** | * | * | * | 9 |
| Veldhuis et al, 2021 | * | * |  | * | ** | * | * | * | 8 |
| Bellan et al, 2022 |  |  | * | * | * | * | * |  | 5 |
| Whiteside et al, 2022 |  |  |  | * | ** |  | * |  | 4 |
| Wang et al, 2024 | * | * | * | * | ** | * | * |  | 8 |
| Wong et al, 2023 | * | * | * | * | ** | * | * |  | 8 |
| Abramoff et al, 2023 |  | * | * | * | ** | * | * |  | 7 |
| Zhang et al, 2022 |  |  | * | * | ** | * | * |  | 6 |
| Taquet et al, 2021 | * | * | * | * | ** | * | * | * | 9 |
| D `Ettorre et al, 2022 |  |  | * | * | ** | * | * |  | 6 |
| Huang et al, 2021 |  |  | * | * | ** | * | * | * | 7 |
| Brito-Zeron et al, 2021 | * | * | * | * | ** | * | * |  | 8 |
| Buonsenso, 2022 |  |  | * | * | ** | * | * | * | 7 |
| Buttery et al, 2021 | * | * | * | * | ** | * | * | * | 9 |
| Cacciatore et al, 2022 | * | * | * | * | ** | * | * | * | 9 |
| Cai et al, 2023 |  |  | * | * | ** | * | * | * | 7 |

Supplementary Table 2. Quality assessment of the included studies using the Newcastle–Ottawa Quality Assessment Scale for case-control studies

|  |  | **SELECTION** | | | **COMPARABILITY** | **Exposure** | | | **Total scores** |
| --- | --- | --- | --- | --- | --- | --- | --- | --- | --- |
| **Author, Year** | Is the case definition adequate?  a) yes, with independent validation *  b) yes, eg record linkage or based on self reports  c) no description | Representativeness of the cases  a) consecutive or obviously representative series of cases *  b) potential for selection biases or not stated | Selection of Controls  a) community controls *  b) hospital controls  c) no description | Definition of Controls  a) no history of disease (endpoint) *  b) no description of source | Comparability of cases and controls on the basis of the design or analysis  a: study controls for age and/or BMI *  b: control for any additional factor*  c: no adjustment or matching | Ascertainment of exposure  a) secure record (eg surgical records) *  b) structured interview where blind to case/control status *  c) interview not blinded to case/control status  d) written self report or medical record only  e) no description | Same method of ascertainment for cases and controls  a) yes *  b) no | Non-Response rate  a) same rate for both groups *  b) non respondents described  c) rate different and no designation |  |
| Gorecka et al., 2022 |  |  | * | * | ** | * | * |  | 6 |
| Azizi et al., 2022 |  |  | * | * | ** | * | * | * | 7 |

Supplementary Table 3. Quality assessment of the included studies using the Newcastle–Ottawa Quality Assessment Scale adapted for Cross-sectional studies.

|  | **SELECTION Selection**  (Maximum 3 stars) | | | **COMPARABILITY**  (Maximum 2 stars) | **Outcome**  (Maximum 3 stars) | | **Total scores** |
| --- | --- | --- | --- | --- | --- | --- | --- |
| **Author, Year** | Representativeness of the sample:  a) Truly representative of the average in the target population.  (all subjects or random sampling) *  b) Somewhat representative of the average in the target population. (non-random sampling) *  c) Selected group of users.  d) No description of the sampling strategy | Non-included subjects:  a) Comparability between respondents and non-respondents’ characteristics is established, and the response rate is satisfactory. *  b) The response rate is unsatisfactory, or the comparability between respondents and non-respondents is unsatisfactory.  c) No description of the response rate or the characteristics of the responders and the non-responders. | Sample size:  a) is justified and satisfactory *  b) Not justified. | The subjects in different outcome groups are comparable, based on the study design or analysis. Confounding factors are controlled.  a) The study controls for the most important factor (select one). *  b) The study control for any additional factor. * | Assessment of the outcome:  a) Independent blind assessment. *  b) Record linkage. *  c) Self report. *  d) No description. | Statistical test:  a) The statistical test used to analyze the data is clearly described and appropriate, and the measurement of the association is presented, including confidence intervals and the probability level (p value). *  b) The statistical test is not appropriate, not described or incomplete. |  |
| Moy, Lim, Hairi et al., 2022 |  |  | * | ** | * | * | 5 |
| Ahmed, Kheder et al., 2021 |  |  | * | ** | * | * | 5 |
| Oliveira, Avila et al., 2022 |  |  | * | ** | * | * | 5 |
| Delgado-Alonso et al., 2022 |  |  |  | ** | * | * | 4 |
| Ferrando et al., 2023 |  |  |  | ** | * | * | 4 |
| Gasnier et al., 2022 |  |  | * | ** | * | * | 5 |
| Goldhaber et al., 2022 | * | * | * | ** | * | * | 7 |
| Goodman et al., 2023 | * | * | * | ** | * | * | 7 |
| Morawa et al., 2023 |  |  | * | ** | * | * | 5 |
| Morioka et al., 2023 | * | * | * | ** | * | * | 7 |
| Nalk et al, 2024 | * | * | * | ** | * | * | 7 |
| Peter et al, 2022 | * | * | * | ** | * |  | 6 |
| Phu et al, 2023 | * | * | * | ** | * | * | 7 |
| Qi et al, 2021 | * | * | * | ** | * | * | 7 |
| Richter et al, 2022 |  |  |  | ** | * | * | 4 |
| Romàn-Montes et al, 2023. |  |  | * | ** | * | * | 5 |
| Tabacof et al, 2022 |  |  | * | ** | * | * | 5 |
| Tebeka et al., 2023 | * | * | * | ** | * | * | 7 |
| Thanh, et al. 2024 | * | * | * | ** | * | * | 7 |
| Walker et al, 2023 | * | * | * | ** | * | * | 7 |

Supplementary Figure 1. Forest plot of pooled prevalence and 95% confidence interval (CI) of (A) Depression (B) Anxiety (C) Composite outcome of Depression and/or Anxiety (D) Stress (E) Suicidality

A

B

C

D

E
