# Supplementary material for: Prevalence of depression, anxiety, stress, and suicide tendency among individual with long-COVID and determinants: A systematic review and meta-analysis
Source: PLoS One. 2025 Jan 28;20(1):e0312351. doi: 10.1371/journal.pone.0312351 (PMC11774403; doi:10.1371/journal.pone.0312351)
Supplement: S2 File — Final. (DOC) [file pone.0312351.s003.doc]

Appendix 2: 1. Characteristics of the excluded studies

| **Row** | **Study** | **Reason for exclusion** |
| --- | --- | --- |
|  | Abd El-Khalik DM, 2023 | The study focuses on individuals with systemic lupus erythematosus (SLE) |
|  | Abdullah S, 2022 | it is not an original research study reporting the prevalence, incidence, or determinants of stress, depression, or suicide tendency in individuals with long COVID. Instead, it focuses on the neuroscience aspects of religious texts and their potential to reduce stress during the post-COVID-19 pandemic era |
|  | Acharya B, 2022 | it focuses on the overall association between the COVID-19 pandemic and suicide rates in Nepal, comparing pre-pandemic and pandemic periods, rather than examining the prevalence, incidence, or determinants of stress, depression, or suicide tendency |
|  | Adeloye D, 2021 | Position Paper |
|  | Aguayo GA, 2023 | it focuses on the association between pre-existing psychotropic medication use and COVID-19 recovery trajectories, rather than specifically examining the prevalence, incidence, or determinants of stress, depression, or suicide tendency in individuals with long COVID after 12 weeks of infection. Additionally, the study focuses on the first 14 days of recovery. |
|  | Ahmed T, 2023 | This study should be excluded because it focuses on the general long-term physical and mental health consequences of COVID-19 in previously hospitalized patients, rather than specifically examining the prevalence, incidence, or determinants of stress, depression, or suicide tendency in individuals with long COVID after 12 weeks of infection. |
|  | Al-Aly Z, 2022 | This study should be excluded because it does not primarily focus on the prevalence, incidence, or determinants of stress, depression, or suicide tendencies related to long COVID after 12 weeks of infection. Instead, it investigates post-acute sequelae in individuals with breakthrough SARS-CoV-2 infections, comparing vaccinated and unvaccinated groups, and the overall risks of various long-term health outcomes beyond mental health. |
|  | Al-Aly Z, 2021 | High-dimensional characterization of post-acute sequelae of COVID-19. It did not present prevalence. |
|  | Alfonsi V,  2023 | Minor population |
|  | Al-Hakeim HK, 2023: Long-COVID post-viral chronic fatigue and affective symptoms are associated with oxidative damage, lowered antioxidant defenses and inflammation: a proof of concept and mechanism study | Did not present prevalence, incidence |
|  | Al-Hakeim HK, 2023: Chronic Fatigue, Depression and Anxiety Symptoms in Long  COVID Are Strongly Predicted by Neuroimmune and  Neuro-Oxidative Pathways Which Are Caused by the  Inflammation during Acute Infection | it does not report the number, prevalence, or incidence of stress, depression, and suicide tendency in individuals with long COVID |
|  | Al-Hakeim HK,  2023: Increased insulin resistance due to long COVID is  associated with depressive symptoms and partly  predicted by the inflammatory response during  acute infection | it focuses on insulin resistance and its association with depressive symptoms in individuals with long COVID, rather than directly reporting the number, prevalence, or incidence of stress, depression, and suicide tendency |
|  | Ali AM, 2021 | it focuses on the development and validation of a shortened version of the Depression Anxiety Stress Scale (DASS) rather than directly reporting the prevalence, incidence, or determinants of stress, depression, and suicide tendency in individuals with long COVID |
|  | Alwaqdani N,  2021 | it specifically focuses on the psychological impact of the COVID-19 pandemic on healthcare workers rather than individuals with long COVID |
|  | Aly MAEG, 2021 | Did not meet COVID 19 definition, S H O R T R E P O R T |
|  | Ambrosetti J, 2021 | ocuses on psychiatric emergency admissions during and after lockdowns rather than specifically on the prevalence of depression, stress, and suicide tendencies among individuals with long COVID |
|  | Anderson KN, 2022 | Report |
|  | Anmella G, 2020 | Correspondence, it focuses on a specific case of a healthcare professional experiencing brief reactive psychosis during the COVID-19 crisis, rather than reporting the prevalence or incidence of stress, depression, and suicide tendencies |
|  | Aolymat I, 2022 | mental health impact of COVID-19 specifically among medical students |
|  | Bai F, 2022 | Did not meet COVID 19 definition, |
|  | Barrea L, 2022 | Review |
|  | Barreto APA,  2022 | Did not meet COVID 19 definition, |
|  | Basheti IA,  2023 | it focuses solely on anxiety and depression among university students in Jordan |
|  | Baumeister A, 2022 | Non-original study |
|  | Becerra-Canales B, 2022 | Non English |
|  | Bechmann N, 2022 | it focuses on sexual dimorphism in COVID-19 and its implications for severity and treatment, without addressing the prevalence or incidence of depression, stress, or suicide tendency |
|  | Bell ML, 2021 | Did not meet COVID 19 definition, |
|  | Bellan M, 2021 | No clear data about outcomes |
|  | Benavente-Fernández A, 2022 | it focuses on hospitalized COVID-19 patients during the initial stage of the pandemic and does not specifically address individuals with long COVID |
|  | Benzakour L,  2022 | Letter to the Editor: Brief Case Report |
|  | Bogale KA, 2023 | it is a qualitative phenomenological |
|  | Bogucki OE,  2023 | it is a case study focusing on the treatment of posttraumatic stress disorder (PTSD) related to COVID-19, |
|  | Bottemanne H, 2021 | it focuses on the relationship between anxiety and depression with persistent physical symptoms (fatigue, dyspnea, and pain) only one month afyter acute infection |
|  | Braga J, 2023 | it focuses on the association between neuroinflammation and persistent depressive and cognitive symptoms (COVID-DC) after COVID-19, rather than directly assessing the prevalence of depression, stress, and suicide tendency |
|  | Brites R,  2023 | it focuses on middle adolescents (aged 16 and 17 years) and their initial psychological reactions to COVID-19 |
|  | Bungenberg J,  2022 | Did not meet COVID 19 definition, |
|  | Carter SJ, 2022 | Did not meet COVID 19 definition, |
|  | Castellini G,  2022 | No data on outcomes |
|  | Chen H,  2022 | No data on outcomes |
|  | Chu Y, 2023 | Minor population |
|  | Ciuluvica Neagu C,  2021 | mental health consequences among dermatologists |
|  | Costa AD, 2022 | No data on outcomes |
|  | Damiano RF, 2022 | No data on outcomes |
|  | Daniel CL, 2023 | Did not meet COVID 19 definition, |
|  | Darcis G, 2021 | No clear data |
|  | Diana L,  2023 | No clear data |
|  | Dixit S, 2022 | it specifically targets college student |
|  | Donnelly SC. 2022 | Non Original study |
|  | Dudine L,  2021 | it focuses specifically on healthcare workers and their experiences |
|  | El Hayek S,  2021 | Did not meet COVID 19 definition, |
|  | El Sayed S,  2020 | No clear data on outcome |
|  | Fernández-de-Las-Peñas C,  2023 | No clear data on outcome |
|  | Fernández-de-Las-Peñas C, 2022 | it primarily focuses on assessing pain-related symptoms in COVID-19 survivors, without specifically reporting on the prevalence of depression, stress, and suicide tendency |
|  | Feter N, 2023 | it focuses on the association between physical activity and the risk of long COVID rather than specifically reporting on the prevalence of depression, stress, and suicide tendency |
|  | Fukase Y, 2023 | it focuses on depression and PTSD among the general population. No clear data on outcome |
|  | Gallagher MW, 2020 | it focuses on the psychological impact of COVID-19 experiences and associated stress on anxiety and depression in the general adult population, rather than specifically examining individuals with long COVID |
|  | Gambaro E, 2021 | it focuses specifically on the mental health of healthcare workers during the COVID-19 pandemic |
|  | Ganesan B,  2021 | primarily focuses on the general population's mental health impacts related to quarantine, isolation, and lockdown measures during the COVID-19 pandemic, rather than specifically on individuals with long COVID |
|  | Gardiner E, 2022 | It does not provide data on the prevalence or incidence of depression, stress, or suicide tendency specifically among those diagnosed with long COVID |
|  | Genecand L, 2023 | Lack of required data |
|  | Gérard M,  2021 | Lack of required data |
|  | Gervasoni F, 2022 | it focuses on the long-term effects of malnutrition and loss of muscle strength in post-COVID patients, without specifically addressing the prevalence or incidence of depression, stress, or suicide tendency |
|  | Lu X,  2021 | Lack of required data |
|  | Magdy R,  2022 | Inor population |
|  | Magnavita N, 2023 | Definition of long COVID not meeting inclusion criteria |
|  | Mahmoudi H, 2021 | Lack of required data |
|  | Malesevic S, 2023 | No clear data |
|  | Margalit I, Y 2022 | it primarily focuses on long-COVID fatigue rather than the prevalence of depression, stress, and suicide tendency |
|  | Mazurek J,  2022 | Lack of required data |
|  | McLoughlin A,  2023 | Lack of required data |
|  | Musetti A,  2023 | No targeted population |
|  | Ng R,  2022 | Lack of required data |
|  | Pagen DME,  2022 | Lack of required data |
|  | Poletti S, 2022 | Lack of required data |
|  | Pandharipande P, 2023 | Lack of required data |
|  | Paul E,  2022 | Definition of long COVID not meeting inclusion criteria |
|  | Parotto M,  2023 | Lack of required data |
|  | Piras I, 2022 | Lack of clear data |
|  | Qattan AMN.  2022 | Do not meet inclusion criteria, targeted population |
|  | Rajapakse T, 2023 | Lack of required data |
|  | Rajkumari B, 2022 | Definition of long COVID not meeting inclusion criteria |
|  | Rastogi R,  2023 | Not targeted population |
|  | Rastogi R, 2023 | Lack of required data |
|  | Pirkis J,  2021 | it analyzes suicide trends related to the early months of the COVID-19 pandemic rather than specifically focusing on individuals with long COVID |
|  | Huang C, 2021 | Retracted article |
|  | Graupensperger S,  2022 | Lack of required data |
|  | Davis HE, 2021 | Definition of long COVID not meeting inclusion criteria, lack of clear data |
|  | Girón Pérez DA, 2022 | Lack of required data |
|  | Carola V,  2022 | Lack of clear data |
|  | Frontera JA, 2022 | Definition of long COVID not meeting inclusion criteria |
|  | Wojahn A, 2023 | Lack of clear data |
|  | Legler F, 2023 | Just in fatigue patients |
|  | Benedetti F, 2021 | Lack of required data |
|  | Taquet M, 2021 | No data on outcome |
|  | Zorn J, 2023 | Lack of required data |
|  | Zhao YJ,  2022 | Lack of required data |
|  | Yang Z, 2022 | Lack of required data |
|  | Xiao Y, 2022: Risks of mental health outcomes in people with covid-19:  cohort study | Definition of long COVID not meeting inclusion criteria |
|  | Xiao Y, 2022: Trends in psychiatric diagnoses by COVID-19 infection and hospitalization among patients with and without recent clinical  psychiatric diagnoses in New York city from March 2020 to  August 2021 | Did not meet inclusion criteria |
|  | Xiao W, 2023 | No long Covid definition |
|  | Wu M, 2022 | Minor population |
|  | Woodward SF, 2022 | Lack of required data |
|  | Wilding S, 2022 | Lack of required data |
|  | Wen M, 2023 | No post covid |
|  | Wei Y, 2023 | Lack of required data |
|  | Wan KS, 2023 | Definition of long COVID not meeting inclusion criteria |
|  | Vuscan ME, 2023 | Lack of required data |
|  | Villalpando JMG,  2022 | Lack of clear definition of long covid |
|  | Van Herck M, 2021 | Lack of required data |
|  | Valero-Bover D, 2022 | Lack of required data |
|  | Vakani K,  2023 | No long covid targeted group |
|  | Uygur OF, 2021 | Definition of long COVID not meeting inclusion criteria |
|  | Tleyjeh IM,  2022 | Lack of required data |
|  | Titi MA, 2022 | Lack of required data |
|  | Thomas-Purcell K, 2023 | Lack of required data |
|  | Thomas M. 2022 | Definition of long COVID not meeting inclusion criteria |
|  | Szlejf C, 2023 | Lack of required data |
|  | Sundarakumar JS,  2023 | No long covid targeted group |
|  | Sugiyama A, 2022 | follow up at a median of 29 days  [IQR 23–128] |
|  | Strawn JR, 2023 | Adolescent group |
|  | Stefano GB. 2021 | Lack of required data, Non original study |
|  | Shachar-Lavie I, 2023 | Lack of required data |
|  | Speichert LJ,  2022 | Definition of long COVID not meeting inclusion criteria |
|  | Sinawi HA, 2021 | No long covid targeted group |
|  | Simonetti A, 2022 | Lack of required data Non original study |
|  | Shivani F,  2022 | Lack of required data |
|  | Shanbehzadeh S, 2023 | Lack of required data |
|  | Sawant N, 2021 | Definition of long COVID not meeting inclusion criteria |
|  | Legler F, 2023 | Lack of required data |
|  | Magnúsdóttir I, 2022 | Lack of required data |
|  | Ryu S,  2022 | Lack of required data |
|  | Saberian P, 2022 | Definition of long COVID not meeting inclusion criteria |
|  | Salfi F, 2023 | Lack of required data |
|  | Samper-Pardo M,  2023 | Lack of required data |
|  | Santiago-Rodriguez EI,  2022 | Do not meet inclusion criteria |
|  | Sara G, 2023 | No long covid |
|  | Schmits E, 2021 | Do not meet inclusion criteria |
|  | Scholz U, 2023 | Lack of required data |
|  | Seto M, 2023 | Minor population |
|  | Sher L. 2020 | No original |
|  | Albott CS, 2020 | Minor population |
|  | Hüfner K, 2023 | Do not meet inclusion criteria, Lack of required data |
|  | Håkansson A, 2020 | Minor population |
|  | Irigoyen-Otiñano M, 2023 | Do not meet inclusion criteria, Lack of required data |
|  | Janiri D, 2020 | Do not meet inclusion criteria, Lack of required data |
|  | Jacobs ET, 2023 | Do not meet inclusion criteria, Lack of required data |
|  | Janiri D, 2022 | Do not meet inclusion criteria, |
|  | Johnson SU, 2022 | Do not meet inclusion criteria, |
|  | Jung YH, 2022 | Non- original |
|  | Juszko K, 2022 | Lack of required data |
|  | Kader N, 2021 | Minor population |
|  | Kaess M, 2023 | Child and adolescent |
|  | Kaltschik S,  2022 | Lack of required data |
|  | Kassir G, 2021 | Lack of required data |
|  | Kataoka M,  2023 | No specifically on Covid 19 |
|  | Kersten J, 2022 | Lack of required data |

2. A table of all data extracted:

Name of data extractors: SB-G and RB-Y

Date of data extraction: November 2023, updated August 2024.

Both these two authors along with other authors confirmed that studies were eligible to be included in the review

| **Author, year** | **Location** | **Study design** | **Study setting** | **Sample size** | **Mean age**  **(years)** | **Male (%)** | **Day zero** | **Severity** | **Follow-up** | **questionnaire** | **Outcome (%)** | **Factors Associated with**  **persistent symptoms *** | **Factors Associated with Depression or Anxiety or suicide, specifically** |
| --- | --- | --- | --- | --- | --- | --- | --- | --- | --- | --- | --- | --- | --- |
| PHOSP-COVID Collaborative Group, 2022 ^1^ | UK | cohort study | Multicentre | 2320 | ≥ 18 years,  58.7 (12·5) | 64·4 | After hospital discharge | ICU | 5 months and 1 year | Generalised Anxiety Disorder 7-item scale [GAD-7]), depression (Patient Health Questionnaire-9 [PHQ-9]) | Depression (25%)  Anxiety (24%) | female sex, obesity, invasive mechanical ventilation | - |
| Abdelrahman MM, 2021 ^5^ | Egypt | cohort study | Single centre | 172 | 17–80 years,  41.8 (17.6) | 34.3% | tested positive | Mixed IP/OP/ICU | 8-10 month | NM | Depression (22%) | Age | - |
| Abramoff BA, et al. 2023 ^7^ | USA | Observational cohort study | Single centre | 324 | 46.6 (14.0) | 31.2 % | Date of the first positive COVID test | Mixed IP/OP/ICU | <12 weeks,  12-29 weeks,  30+ weeks | The hospital anxiety scale and Hospital depressions cale | Severe Depression (36%)  Severe Anxiety (44%) | Poor and African American/ Black individuals | Poor and African American/ Black individuals |
| Ahmed GK, 2021 Egypt ^16^ | Egypt | cohort study | Single centre | 182 | 46.49 (17.4) | 46.2% | Date of the first positive COVID test | IP/ ICU | 6 months | Symptom checklist 90 "SCL 90′′ | Depression (11.5%)  Anxiety (28%) | being female, diabetes, oxygen support or mechanically ventilated | being female, diabetes, oxygen support or mechanically ventilated |
| Azizi A, et al. 2022 ^48^ | Morocco | case-control study | Single centre | 213 | 55.1 (16.4) | 53.1 % | After hospital discharge | IP/ ICU | 3 months | The Hospital Anxiety and Depression Scale (HADS) | Depression (10.9%)  Anxiety (12.8%) | Older patients, suffering from type 2 diabetes and kidney diseases, admitted to ICU, who stayed a  long duration in the hospital, who had severe and longer duration of symptoms and who used Chloroquine | Older patients, suffering from type 2 diabetes and kidney diseases, admitted to ICU, who stayed a  long duration in the hospital, who had severe and longer duration of symptoms and who used Chloroquine |
| Becker C, et al. 2021 ^64^ | Switzerland | prospective cohort study | Single centre (2 hospitals) | 90 | 60 (15.1) | 62 % | After hospital discharge | IP/ ICU | 1 year | The Hospital Anxiety and Depression Scale (HADS) | Depression (10%) Anxiety (13%)  Stress (18%)  Depression and/or Anxiety (18%) | duration of hospitalization, severity of illness, self-perceived overall health status 30 days after hospitalization | - |
| Bellan M, et al. 2022 ^67^ | Italy | prospective cohort study | Single centre | 324 | 60 (50-69)** | 60.5 % | After hospital discharge | IP/ ICU | One year | interviewed by an experienced psychiatrist | Depression (32%)  Anxiety (23%) | diffusing capacity of the lungs for carbon monoxide (DLCO) (> 80%), | - |
| Brito-Zerón P, et al. 2021 ^89^ | Spain | cohort study | Multi centre | 38 | 54.3 (13.3) | 5.3 % | Date of the first positive COVID test | Mixed IP/OP/ICU | 5 months | NM | Depression and/or Anxiety (59%) | raised LDH levels, raised CRP levels, use of hydroxychloroquine and antiviral agents, hospital admission, mean length of hospital admission and requirement of supplemental oxygen | - |
| Buonsenso D, et al. 2022 ^94^ | Italy | retrospective cohort study | Single centre | 155 | 46.48 (7.3) | 49.7% | Date of the first positive COVID test | Mixed IP/OP/ICU | within one year of the  acute illness | Ad-Hoc Questionnaire | Anxiety (45.1%) | NM (low quality) | - |
| Buttery S, et al. 2021 ^97^ | UK | mixed methods approach, both quantitative  and qualitative methods | Nationwide | 2506 | (17 or under to 75 or older) | 22% | Date of the first positive COVID test | Mixed IP/OP/ICU | (8-12) weeks and >  12 weeks | NM | Depression and/or Anxiety (45%) | NM | - |
| Cacciatore M, et al. 2022 ^98^ | Italy | secondary analysis of a cohort study | Single centre | 83 | 66.9 (64.2-69.7)** | 75.9% | After hospital discharge | IP/ ICU | Within six months after discharge with mean of 3.5 months | The Hospital Anxiety and Depression Scale (HADS) | Depression (15.7%)  Anxiety (14.5%) | NM | - |
| Cai J, et al. 2023 ^99^ | China | Cohort study | Single centre | 21799 | 43.8 (13.6) | 64.47 % | After hospital discharge | IP/ ICU | 6 months and  12 months | Generalized  Anxiety Disorder-7 (GAD-7) questionnaire, Patients  Health Questionnaire (PHQ-9) | Depression (41.9%)  Anxiety (34.4%) | Females, youth (age <40 years), middle age (40–60 years), ≥ 2 comorbidities, severe infection in the acute phase | - |
| Calabria M, et al. 2022 ^100^ | Spain | cross-sectional study | Single centre | 136 | 51.7 (13.5);  range (20–88) | 36% | Date of the first positive COVID test | Mixed IP/OP/ICU | 8 months | Hospital Anxiety  and Depression Scale (HADS | Depression (23.5%)  Anxiety (35.3%) | NM | - |
| Caspersen IH, et al. 2022 ^108^ | Norway | Cohort study | Multi centre | 774 | NM | 42.0 % | Date of the first positive COVID test | Mixed IP/OP/ICU | 12 months | Ad-Hoc Questionnaire | Depression (4.1%)  Anxiety (3.5%) | NM | - |
| Catalán IP, et al. 2022 ^111^ | Spain | Cohort study | Single centre | 76 | (≥18 years) | 75 % | After hospital discharge | IP/ ICU | 12 months | SF‐36 quality  of life questionnaire | Depression (14.4%)  Anxiety (23.3%) | NM | - |
| Clemente I, et al. 2022 ^126^ | Italy | Cohort study | Single centre | 48 | 62.5 (10.1) (range: 40-85) | 70.8% | After hospital discharge | IP/ ICU | 3 months | Symptom  Checklist-90 (SCL90) | Depression (29.2%)  Anxiety (25%) | Female | - |
| Damiano RF, et al. 2023 ^152^ | Brazil | cohort study | single center | 710 | 55 (14.1) | 52% | After hospital discharge | IP/ ICU | 6-11 month | Hospital Anxiety and Depression Scale (HAD) | Depression (7.4%)  Anxiety (14.6%) | WHO severity, Comorbidity, socio-demographic variables of sex, older age, and lower education level. | Female, lower educational level, Older age |
| Danesh V, et al. 2023 ^153^ | USA | cohort study | Multi centre | 441 | 51.5 | 33.1% | Date of the first positive COVID test | Mixed IP/OP/ICU | 9 month | A semi-structured interview | Depression (5.8%)  Anxiety (11.5) | NM | - |
| de Miranda DAP, et al. 2022 ^159^ | Brazil | longitudinal study | single center | 324 | 42.6% were aged 41–60, 31.5% 21–40, 21.3% 61–80 | 36 .7 % | Date of the first positive COVID test | Mixed IP/OP/ICU | 14-months | NM | Anxiety (8%) | Age, severity of SARS-CoV-2 acute infection, presence comorbidities | - |
| de Oliveira JF, et al. 2022 ^160^ | Brazil | cross-sectional study | single center | 369 | 57 (46-66) ** | 49 % | After hospital discharge | IP/ ICU | 2-12 months | EuroQol Group Association five-domain, three-level questionnaire (EQ-5D-3L) | Depression and/or Anxiety (55.1%) | ICU admission, Dysgeusia | - |
| Delgado-Alonso C, et al. 2022 ^163^ | Spain | Cross-sectional study | single center | 50 | 51.0 (11.6) | 26 % | Date of the first positive COVID test | Mixed IP/OP/ICU | Minimum 3 months Mean: 9.1 (3.4) months | State-Trait Anxiety Inventory (STAI) and Beck Depression Inventory-II | Depression (30%)  Anxiety (52%) | NM | - |
| d'Ettorre G, et al. 2022 ^802^ | Italy | longitudinal cohort study | Single center | 137 | 18–45: 14.6%  46–70: 75.9%  71–85: 9.5% | 53.3 | After hospital discharge | IP/ ICU | 2 years | The EuroQol 5-Dimensions | Depression and/or Anxiety (51.1%) | Female gender, unemployed  status, and chronic comorbidities | - |
| Egger M, et al. 2024 ^22 update^ | Germany | Observational prospective cohort study | Single center | 97 | 61 (12) | 69% | post-discharge | ICU | 3, 6, and 12 months | Hospital Anxiety and Depression Scale (HADS), | Depression (40%)  Anxiety (42%) | Mechanical Ventilation, Preclinical Frailty, Obesity | Suffering from fatigue, Mechanical Ventilation, Obesity |
| Fancourt D, et al. 2023 ^194^ | UK | longitudinal study | Multi centre | 495 | 57.3 | 21.8% | Date of the first positive COVID test | Mixed IP/OP/ICU | over 22 months. | Patient Health Questionnaire (PHQ-9), Generalized Anxiety Disorder assessment (GAD-7) | Depression (16%)  Anxiety (35%) | NM | - |
| Fernández-de-Las-Peñas C, 2022 ^206^ | Spain | longitudinal study | Multi centre | 1969 | 61 (16) | 53.5% | After hospital discharge | IP/ ICU | mean of 8.4 (1.5) months | Hospital Anxiety and  Depression Scale (HADS) | Depression (19%)  Anxiety (15.7) | aged 60–70 years, | - |
| Fernández-de-Las-Peñas et al. 2024 ^19 update^ | Spain | Multicenter longitudinal study | Multicenter | 1266 | 61 (16) | 54,4% | After hospital discharge | Mixed IP/ICU | 6,12 and 18 months | Hospital Anxiety and Depression Scale (HADS-A and HADS-D) | Depression (22%)  Anxiety (14.6%) | No specific risk factors identified | No specific risk factors identified |
| Ferrando SJ,et al. 2023 ^208^ | USA | Cross-sectional study | single center | 75 | 43.5 | 29.3% | Date of the first positive COVID test | Mixed IP/OP/ICU | Mean of 220 days | generalized anxiety  questionnaire-7 (GAD-7) | Anxiety (31%) | NM | - |
| Frontera JA, et al. 2022 ^217^ | USA | prospective, longitudinal cohort study | Multi centre | 451 at 6-month and 383 at  12-month | At 6 months: 69 (57–78)**, at 12 months: 65 (53–73)** | 65% | post diagnosis | Mixed IP/OP/ICU | 6 and 12-months | Quality of Life in Neurological Disorders (NeuroQoL) | Depression (11%)  Anxiety (12%) | life stressors including  financial insecurity, food insecurity, death of a close contact and new disability, older age, female sex, index COVID-19 severity | life stressors including  financial insecurity, food insecurity, death of a close contact and new disability, older age, female sex, index COVID-19 severity |
| Frontera JA, et al. 2021 ^219^ | USA | prospective, longitudinal cohort study | Multi centre | 395 | 68 (55–77) ** | 65% | post diagnosis | Mixed IP/OP/ICU | 6 months | Quality of Life in  Neurological Disorders (Neuro-QoL) | Depression (25%)  Anxiety (46%) | patients with neurological complications during index hospitalization | patients with neurological complications during index hospitalization |
| Garout MA, et al. 2022 ^228^ | Saudi Arabia | Cross-sectional study | single center | 744 | 18–29, 138 (18.6%)  30–50, 398 (53.5%)  ≥ 50, 207 (27.9%) | 49.3% | Date of the first positive COVID test | Mixed IP/OP/ICU | Less than 3 months, 3-6 months, after 6 months | COVID‐19 Yorkshire Rehabilitation Screening  (C19‐YRS) | Depression (9.5%)  Anxiety (13.2%) | NM | - |
| Gasnier M, et al. 2023 ^229^ | France | Cross-sectional study | single center | 177 | ≥18 years,  mean; 56 years | 41.1% | After hospital discharge | ICU | 4 months | Hospital Anxiety and Depression scale-Anxiety  subscale (HAD-A), Beck Depression  Inventory-13  items (BDI) and Mini International Neuropsychiatric Interview  (MINI 5.0) | Depression (13.6%)  Anxiety (11.3%) | Respiratory complaints, Number of long COVID complaints | Respiratory complaints, Number of long COVID complaints |
| Gil S, et al. 2021 ^236^ | Brazil | cohort study | single center | 614 | 56 (13) | 53% | After hospital discharge | IP/ ICU | 6 to 11 months | NM | Depression (16.2%)  Anxiety (16.2%) | physical inactivity | - |
| Goldhaber NH, et al. 2022 ^240^ | USA | Cross-sectional study | Multi centre | 421 | (range: 18–89 years)  52.2 (15) | 63.4% | Date of the first positive COVID test | Mixed IP/OP/ICU | 331 days (range: 120–617; SD = 84) | Generalized Anxiety Disorder 2-item  (GAD-2) and the Patient Health Questionnaire 2-item (PHQ-2) | Depression (28.3%)  Anxiety (47%) | female sex, COVID-19 hospitalization, poorer  pre-COVID self-rated health, younger age | Younger age |
| Goodman ML, et al. 2023 ^242^ | USA | cross-sectional population survey | Multi centre | 621 | (range : 20–50  Year)  Mean : 36.9 | 16.5% | Date of the first positive COVID test | Mixed IP/OP/ICU | After 3 month | self-administered  questionnaire | Suicidality (21.2%)  Depression (28.3%)  Anxiety (47.1%) | higher daily functional challenges  and common mental disorders | - |
| Gorecka M, et al. 2022 ^243^ | UK | Prospective case–control study | single center | 20 | 45 (13) | 47% | Date of the first positive COVID test | Mixed IP/OP/ICU | Afetr 12 weeks | EuroQol Five-Dimensional Five-Level questionnaire (EQ-5D-5L) | Depression and/or Anxiety (50%) | NM | - |
| Gramaglia C, et al. 2022 ^245^ | Italy | cohort study | single center | 200 | 61.5 (51.0-  70.5) ** | 61.2% | After hospital discharge | IP/ ICU | 1 year | Beck Depression Inventory, Beck Anxiety Inventory | Depression (24.4%)  Anxiety (16.8) | female gender and depressive  symptoms at 4-months follow-up, arterial hypertension, obesity, | female gender and depressive  symptoms at 4-months follow-up, arterial hypertension, obesity, |
| Guo Y, et al. 2023 ^255^ | China | prospective cohort study | Multi centre | 208 | 58 (50.0- 64.3) ** | 48.1% | Date of the first positive COVID test | Mixed IP/OP/ICU | 3.3 months,  9.2 months, 18.5 months | Patient Health Questionnaire-9 (PHQ-9) | Depression (55.2%)  Anxiety (37.1%) | NM | - |
| Han JH, et al. 2022 ^261^ | USA | prospective cohort study | Multi centre | 213 | 45 (33, 57) ** | 34.8 % | Date of the first positive COVID test | Mixed IP/OP/ICU | 6-11 months | EuroQol  visual analogue scale, EuroQol Five-dimensional Five-Level questionnaire Patient Health Questionnaire | Depression and/or Anxiety (40%) | poorer long-term health status, poorer quality of life, and psychological distress. | - |
| Hastie CE, et al. 2022 ^267^ | UK | prospective cohort study | Multi centre | 33281 | >16 years  45  (31–56) ** | 39% | Date of the first positive COVID test | Mixed IP/OP/ICU | 6, 12 and 18-month | EuroQol-5 Dimension (EQ-5D) | Depression and/or Anxiety (18.8%) | hospitalized infection, ICU admission, age,  female sex, deprivation, respiratory disease, and multimorbidity | - |
| Hellemons ME, et al. 2022 ^277^ | Netherlands | prospective cohort study | single center | 92 | 58.2 (12.3) | 63.0% | After hospital discharge | IP/ ICU | 3 and 6  months | Hospital Anxiety and  Depression Scale (HADS) | Depression (16.1%)  Anxiety (14%) | NM | - |
| Herman B, et al. 2022 ^279^ | Indonesia | prospective cohort study | single center | 712 | NM | 40.3% | Date of the first positive COVID test | Mixed IP/OP/ICU | 60 days | Patient Health Questionnaire-9 (PHQ-9) | Depression (18.5%) | Favipiravir prescription, patients living alone | Favipiravir prescription, patients living alone |
| Holdsworth DA, et al. 2022 ^280^ | UK | prospective cohort study | single center | 205 | 39  (30-46.7) ** | 84% | Date of the first positive COVID test | Mixed IP/OP/ICU | 24 week (IQR17.1–34.0) | Generalized anxiety disorder-7, GAD-7),  patient health questionnaire 9 | Depression (24%)  Anxiety (17%) | NM | - |
| Houben-Wilke S, et al. 2022 ^283^ | Netherlands | prospective cohort study | single center | 239 | 50  (39-56) ** | 18 % | Date of the first positive COVID test | Mixed IP/OP/ICU | 3 and 6 months | Hospital Anxiety and Depression Scale [HADS] | Depression (46.9%)  Anxiety (35.6%) | NM | - |
| Huang L, et al. 2022 ^288^ | China | longitudinal cohort study | single center | 2469 | 57·0 (48·0–65·0) ** | 54% | After hospital discharge | IP/ ICU | 6, 12 and 24 months | Generalized Anxiety Disorder  seven-item scale (GAD-7), the Patient Health  Questionnaire 9 (PHQ-9) | Depression and/or Anxiety (23%) | NM | - |
| Huang L, et al. 2021 ^815^ | China | longitudinal cohort study | Single center | 1276 | 59·0 ( 49–67)** | 53 | After hospital discharge | IP/ ICU | 6 and 12 months | The EuroQol 5-Dimensions five-level (EQ-5D-5L) | Depression and/or Anxiety (23%) | Female gender | Female gender |
| Jiménez-Rodríguez BM, et al. 2022 ^302^ | Spain | longitudinal cohort study | single center | 217 | 59 (49–68) ** | 53.5% | Date of the first positive COVID test | Mixed IP/OP/ICU | 2 and 6 months | NM | Depression (57.1%) | NM | - |
| Jawad MJ, et al. 2021 ^2^ | IRAQ | cross-sectional study | Multicentre | 200 | NM | 66.5% | tested positive | NM | 6 months | Patient Health Questionnaire and State Trait Anxiety Inventory | Depression (55%)  Anxiety (44%) | male gender, | male gender |
| Kayaaslan B, et al. 2021 ^316^ | Turkey | longitudinal cohort study | single center | 1007 | 18–34 : (33.1%)  35–49 : (27.0%)  ≥ 50 : (39.9%) | 54.4% | Date of the first positive COVID test | Mixed IP/OP/ICU | beyond 12 weeks | Ad-hoc questionnaire | Depression (1%)  Anxiety (1.5%) | Severe acute COVID‐19, hospitalization, and presence of comorbidity | - |
| Kim Y, et al. 2023 ^331^ | Republic of Korea | prospective online surveys | single center | 132 | (range: 16–70 years)  38.0 (24.0-50.5) ** | 31.8% | Date of the first positive COVID test | Mixed IP/OP/ICU | 6, 12, and 24 months | EuroQol-5 dimension (EQ5D) | Depression (25%)  Anxiety (24%) | COVID-19 vaccination or the number of vaccinations received may not significantly affect the  incidence of long COVID | - |
| Kim Y, et al. 2022 ^332^ | Republic of Korea | prospective online survey | single center | 127 | (range: 17–70 years)  37 (26.0–51.0) ** | 32% | Date of the first positive COVID test | Mixed IP/OP/ICU | 6 and 12 months  454 [IQR] 451–458) days | EuroQol-5 dimension (EQ5D) | Depression (24.9%)  Anxiety (24.1%) | Older age, female sex, and disease severity | Older age, female sex, and disease severity |
| Kim Y, et al. 2022 ^333^ | Republic of Korea | prospective cohort study | single center | 170 | 51 (37–61) ** | 40% | Date of the first positive COVID test | Mixed IP/OP/ICU | 6 and 12 months | the Patient Health  Questionnaire-9 (PHQ-9), Generalized Anxiety  Disorder-7 (GAD-7) | Depression (20%)  Anxiety (24.1%) | NM | - |
| Koliadenko N. Vm et al. 2022 ^336^ | Bangladesh | prospective cohort study | single center | 129 | (range: 20-89)  20-29: 12.4%  30-39: 13.2%  40-49: 25.6%  50-59: 27.9%  60-69: 11.6%  70-79: 7%  80-89: 2.3% | 47.2% | Date of the first positive COVID test | Mixed IP/OP/ICU | 10 months | Depression, Anxiety, and Stress Scale-21 (DASS-21) | Depression (16.3%) Anxiety (20.9%)  Stress (21.7%) | NM | - |
| Kruger A, et al. 2022 ^339^ | South Africa | Cross-sectional study | single center | 99 | NM | 30.3% | Date of the first positive COVID test | Mixed IP/OP/ICU | After 3 months | NM | Depression and/or Anxiety (30%) | NM | - |
| Kucukkarapinar M, et al. 2022 ^342^ | Turkey | prospective cohort study | single center | 90 | 44.58 (15.36) | 45.2% | Date of the first positive COVID test | Mixed IP/OP/ICU | After 6 months | Depression, Anxiety and Stress Scale-21 (DASS-21) | Depression (32%) Anxiety (21.1%)  Stress (33.3%) | NM | - |
| Li D, et al. 2022 ^358^ | China | prospective cohort study | single center | 155 | 43 (34–55) ** | 52.3% | After hospital discharge | IP/ ICU | up to 2 years | Generalized Anxiety  Disorder 7-item (GAD-7) scale,  Patient Health Questionnaire-9 (PHQ-9), | Depression (9.1%)  Anxiety (8.4%) | NM | - |
| Martino GP, et al. 2022 ^399^ | Italy | prospective cohort study | single center | 64 | 68 | 64% | After hospital discharge | IP/ ICU | 6 and 12 months | NM | Depression (56.4%)  Anxiety (48.5%) | severe COVID-19 | - |
| Martínez-Cao C, et al. 2021 ^1004^ | Spain | prospective cohort study | International | 5638 | 18–29:  8.78%  30–39: 23.04%  40–49:  29.07%  50–59:  24.02%  60–69:  11.56%  70–79:  3.25%  80+:  0.28% | 19.97% | Date of the first positive COVID test | Mixed IP/OP/ICU | More than 2 months  Median: 190 days (164–229 days) | Patient Health Questionnaire-2 (PHQ-2), Generalized  Anxiety Disorder scale-7 (GAD-7) | Depression (32.7%)  Anxiety (25.2%) suicidality (17.2%) | younger age, greater reductions in overall health, higher symptom severity, limitations to physical capability, lower income, financial hardship, psychiatric history, employment impact, male sex, men and non-binary gender, and negative experiences with  medical professionals, family, friends, partners and employers | younger age, greater reductions in overall health, higher symptom severity, limitations to physical capability, lower income, financial hardship, psychiatric history, employment impact, male sex, men and non-binary gender, and negative experiences with  medical professionals, family, friends, partners and employers |
| Mazza MG, et al. 2021 ^409^ | Italy | prospective cohort study | single center | 246 | 60.1 (12.2) | 69% | After hospital discharge | IP/ ICU | 6 and 12 months | State-Trait Anxiety Inventory, Zung Severity Rating Scale | Depression (25%)  Anxiety (32%) | Gender | Female gender |
| Mazza MG, et al. 2021 ^410^ | Italy | prospective cohort study | single center | 226 | (range:26 to 87 y)  58.5 (12.7) | 77% | After hospital discharge | IP/ ICU | 3 months (90.1 ± 13.4 days) | Zung Self-  Rating Depression Scale (ZSDS), 13-item Beck’s Depression  Inventory (BDI-13), State-Trait Anxiety Inventory | Depression (40%)  Anxiety (31%) | Females, patients with a positive  previous psychiatric diagnosis, Duration of hospitalization, presence of psychopathology at one month after discharge | Females, patients with a positive previous psychiatric diagnosis, Duration of hospitalization, presence of psychopathology at one month after discharge |
| Mendola M, et al. 2022 ^418^ | Italy | prospective cohort study | single center | 56 | 55  (50-61.2) ** | 50% | After hospital discharge | IP/ ICU | 18 months | Ad-hoc questionnaire | Anxiety (8.8%) | NM | - |
| Menges D, et al. 2021 ^419^ | Switzerland | population-based prospective cohort study | National wide | 431 | 47 (33 to 58) ** | 50.3% | Date of the first positive COVID test | Mixed IP/OP/ICU | 6-8 months  Median: 7.2 months (range 5.9-10.3 months) | 21-item Depression, Anxiety and Stress Scale (DASS-  21) | Depression (25.9%) Anxiety (31.7%)  Stress (16%)  Depression and/or Anxiety (47%) | NM | - |
| Morawa E, et al. 2023 ^426^ | Germany | prospective cohort study | single center | 110 | 42.5 (11.9) | 31.8% | Date of the first positive COVID test | Mixed IP/OP/ICU | More than 3 months  Mean (SD) (13.5 (8.3) | Patient-Health-Questionnaire-9  (PHQ-9) | Depression (68.8%) | NM | - |
| Morioka S, et al. 2023 ^428^ | Japan | cross-sectional questionnaire-based survey | Single center | 502 | 48.0  (42.0-55.0) ** | 40.2 | Date of the first positive COVID test | Mixed IP/OP/ICU | 6, 12, 18, and 24 months | Ad-hoc questionnaire | Depression (13.9%) | being female, moderate or severe COVID-19, underlying medical conditions, younger age | younger age |
| Moy et al. 2022 ^1001^ | Malaysia | cross-sectional online  questionnaire study | Single center | 598 | 40.2 (10.9) | 41.3 | Date of the first positive COVID test | Mixed IP/OP/ICU | 12 weeks | Patient Health Questionnaire 9 (PHQ-9) | Depression (47.3%) | Female, younger age, being overweight/obese, perceiving to have poorer health | Female, younger age, being overweight/obese, perceiving to have poorer health |
| Naik H, et al. 2024 ^40 update^ | USA | Cross-sectional Study | Nationwide | 844 | 46 | 49.8% | COVID-19 diagnosis date | Mixed IP/OP/ICU | 3 months | Patient Health Questionnaire-8 (PHQ-8) and General Anxiety Disorder-7 (GAD-7) | Depression (16.8%)  Anxiety (16.7%) | NM | NM |
| Ocsovszky Z, et al. 2022 ^449^ | Hungary | prospective cohort study | single center | 166 | 39.06 (14.49) | 43.4 | Date of the first positive COVID test | Non-hosptalized OP | mean 25 ± 18 weeks | Beck-Depression Inventory (BDI), Beck Anxiety Inventory (BAI) | Depression (73.5%)  Anxiety (12%) | Depression symptoms during acute infection age, and life, satisfaction, presence of pre-existing affective or  anxiety problems | pre-existing mental health problems |
| O’Kelly B, et al. 2022 ^452^ | Ireland | prospective cohort study | single center | 155 | 43.3 (31-52) | 32 | Date of the first positive COVID test | Mixed IP/OP/ICU | 2-4 and 7-14 months | SF-12 Health Survey (SF-12) | Anxiety (2%) | number of initial symptoms | - |
| Orrù G, et al. 2021 ^457^ | Italy | cross-sectional questionnaire-based survey | Single center | 507 | <20 : 0.20%  20–29: 12.23%  30–39: 20.91%  40–49: 30.77%  50–59: 26.04%  60–69: 8.28%  >70: 1.58% | 17.9 | Date of the first positive COVID test | Mixed IP/OP/ICU | More than 3 months | EuroQol Five-Dimensional Questionnaire: EuroQol-  5D (EQ-5D) | Depression (22.7%)  Anxiety (34.2%) | NM | - |
| Peter RS, et al. 2022 ^486^ | Germany | Population based, cross sectional study | National wide | 12053 | (range: 18-65 years) 44.1 | 41.2 (13.7) | Date of the first positive COVID test | Mixed IP/OP/ICU | 6 to 12 months, mean: 8.5 months | SF-12 Health Survey (SF-12) | Depression and/or Anxiety (21.1%) | NM | - |
| Phu DH, et al. 2023 ^487^ | Thailand | cross-sectional study | Multi center | 939 | 19–59: 84.7%  ≥ 60: 15.3% | 22.6 | Date of the first positive COVID test | Mixed IP/OP/ICU | After 3 months | 21-item Depression Anxiety and Stress Scale  (DASS-21) | Depression (11.1%), Anxiety (19.1%)  Stress (4.8%) | female patients, medical history, low income, | female patients, medical history, low income, |
| Qi T, er al. 2021 ^504^ | China | cross-sectional- online survey study | Single center | 1171 | Male:  31.66 female: 34.61 | 42.19% | Date of the first positive COVID test | Mixed IP/OP/ICU | more than 1 year | patient health questionnaire-9 (PHQ-9) and generalized anxiety disorder-7 (GAD-7) | Depression (22.6%)  Anxiety (24.1%) | Living alone, regular exercises (protective), negative attitude towards the pandemic | Living alone, regular exercises (protective), negative attitude towards the pandemic |
| Richter D, et al. 2022 ^525^ | Germany | cross-sectional- study | Single center | 70 | 50.5 (40–58)** | 31.4 | Date of the first positive COVID test | OP | more than 3 months | Hospital Anxiety and Depression Scale (HADS) | Depression (28%)  Anxiety (42%) | Hypoechogenic brainstem raphe alterations in transcranial sonography (TCS) | Hypoechogenic brainstem raphe alterations in transcranial sonography (TCS) |
| Román-Montes CM, et al. 2023 ^530^ | Mexico | cross-sectional study | Single center | 246 | 50 (41–63) ** | 54.87 | After hospital discharge | IP/ ICU | After 3 months, 150 days (IQR 90–225) | EuroQol Five-Dimensional Questionnaire: EuroQol-  5D (EQ-5D) | Depression and/or Anxiety (58%) | Women, tobacco smoking, severity of lung involvement in the initial chest tomography | - |
| Samper-Pardo M, et al. 2023 ^544^ | Spain | secondary data analysis from a randomized clinical trial | Single center | 100 | (range: 29–72), 48.2 (9.2) | 20% | Date of the first positive COVID test | Mixed IP/OP/ICU | After 3 months, Median of 18 months | Hospital Anxiety and Depression Scale (HADS) | Depression and/or Anxiety (8.3%) | Educational level, number of persistent symptoms, affective affectation, | Educational level, number of persistent symptoms, affective affectation |
| Sayde GE, et al. 2023 ^554^ | USA | prospective cohort study | single center | 77 | 68 (63-73) ** | 95.2% | After hospital discharge | IP/ ICU | 3 and 6 months | 9-question Patient Health Questionnaire (PHQ-9), and 7-item Generalized Anxiety Disorder (GAD-7). | Depression (15.9%)  Anxiety (6.8%) | NM | - |
| Schandl A, et al. 2021 ^556^ | Sweden | prospective cohort study | single center | 113 | NM | 76.1% | After hospital discharge | ICU | Mean of 5 months | Hospital anxiety and depression scale (HADS) | Depression (36%)  Anxiety (33%) | NM | - |
| Spada MS, et al. 2022 ^593^ | Italy | prospective cohort study | single center | 1457 | 59.4 (13.7) | 62.5% | After hospital discharge | Mixed IP/OP/ICU | After 3 months (97.6 ±  48.1 days) | Hospital Anxiety and Depression Scale (HADS) | Depression (5.9%)  Anxiety (14.2%) | hospitalization – regardless of the setting of care – and promptness in  follow-up evaluation had protective effect. | hospitalization – regardless of the setting of care – and promptness in  follow-up evaluation had protective effect. |
| Stallmach A, et al. 2022 ^597^ | Germany | prospective cohort study | single center | 355 | (range: 17–86), 51 (40, 60) ** | 40% | Date of the first positive COVID test | Mixed IP/OP/ICU | 6 months | Patient Health Questionnaire, PHQ-9 | Depression (10.9%) | hospitalization, ICU admission, | hospitalization, ICU admission, |
| Staudt A, et al. 2022 ^598^ | Germany | prospective cohort study | single center | 101 | 60 (range 28–69) | 58% | After hospital discharge | OP/ICU | 10 months | Patient Health Questionnaire, PHQ-9 | Depression (20%) | fatigue, cognitive impairment and low quality of life | fatigue, cognitive impairment and low quality of life |
| Tabacof L, et al. 2022 ^617^ | USA | cross-sectional  study | Single center | 156 | 44 (13–79) ** | 31% | Date of the first positive COVID test | Mixed IP/OP/ICU | median  351(82–457 days) | generalized anxiety disorder scale (GAD-7), patient health questionnaire-2 (PHQ-2), EuroQol Five-Dimensional Questionnaire: EuroQol-  5D (EQ-5D) | Depression (8%), Anxiety (12%), Stress (89%) | NM | - |
| Talhari C, et al. 2023 ^621^ | Brazil | cross-sectional online survey | Multi center | 5791 | < 30:  20.8%  31-60:  67.6%  > 60:  11.6 | 25.4 | Date of the first positive COVID test | Mixed IP/OP/ICU | 12 weeks | Ad-hoc questionnaire | Depression (25%)  Anxiety (31.2%) | Female sex, myalgia, anosmia, and  severe disease, Pre-existing  depression | Pre-existing  depression |
| Taquet M, 2021 ^626^ | UK | retrospective cohort study | Nationwide | 273618 | 46.3 (19.8) | 43.4% | Date of the first positive COVID test | Mixed IP/OP/ICU | 6 months | NM | Depression and/or Anxiety (15.5%) | age, sex, or severity of infection | - |
| Titze-de-Almeida R, et al. 2022 ^641^ | Brazil | prospective cohort study | single center | 236 | (range: 19-82 y) 41.2 (12.8) | 39% | Date of the first positive COVID test | Mixed IP/OP/ICU | 5–8 months | Generalized Anxiety Disorder  2-item questionnaire (GAD-2) | Depression (44.9%)  Anxiety (36.9%) | NM | - |
| Thanh HN, et al. 2024 ^38 update^ | Vietnam | Cross-sectional Study | Multicenter | 394 | 29.46 (12.17) | 36.3% | NM | Mixed IP/OP/ICU | 3 months | Depression, Anxiety, and Stress Scale (DASS-21) | Depression (28.7%), Anxiety (26.4%), Stress (20.6%) | Female gender and quality of life issues | NM |
| Tebeka S, et al. 2023 ^update7^ | France | Cross-sectional survey | National wide | 1095 | 18–24: 9.8%  25–34: 27.8%  35–44: 19.5%  45–54: 17.7%  55–64: 14.0%  ≥65: 11.3% | 39% | COVID-19 diagnosis date | Mixed IP/OP/ICU | 3 months | Generalized Anxiety Disorder (GAD-2), Patient Health Questionnaire (PHQ-2) | Depression (8%)  Anxiety (12%)  Stress (69%) | Chronic anxiety | Pre-existing chronic anxiety |
| Tsai J, et al. 2024 ^27 update^ | USA | Longitudinal cohort study | Multicenter | 3595 (511 completed follow-up over 6 months) | 38.52 | 20.41% | day of laboratory-confirmed COVID-19 infection | Mixed IP/OP/ICU | 3 months, and 6 months | Patient Health Questionnaire-4 | Depression (5%) | Being white hispanic ethnicity, older age, Greater Anxiety and Depression Symptoms | Greater Anxiety and Depression Symptoms at beginning |
| Taquet M, eta l. 2021 ^715^ | UK | retrospective cohort study | National wide | 236 379 | 46 (19·7) | 44% | Date of the first positive COVID test | Mixed IP/OP/ICU | 6 months | NM | Anxiety (17.4%) | severe COVID-19 | severe COVID-19 |
| Veldhuis CB,et al. 2021 ^661^ | USA | prospective cohort study | National wide | 1567 | 18–30: 38.0%  31–40: 31.8%  41–50: 14.2%  51–65: 12.3%  66+:  3.7% | 11.8% | Date of the first positive COVID test | Mixed IP/OP/ICU | 5 months | Epidemiologic  Studies Depression (CES-D), Generalized Anxiety Disorder 7-item  (GAD-7) scale | Depression (55%)  Anxiety (65%) | Specific demographic groups (people of color and sexual and gender minorities) | Specific demographic groups (people of color and sexual and gender minorities) |
| Walker S, et al. 2023 ^667^ | UK | cross-sectional  study | National wide | 3754 | 47.7 (12.3) | 28.2 | Date of the first positive COVID test | Mixed IP/OP/ICU | 12 weeks or more | Patient  Health Questionnaire–Eight Item Depression Scale), (Generalised Anxiety Disorder Scale, Seven-Item | Depression (61.1%)  Anxiety (74.2%)  Depression and/or Anxiety (95%) | NM | - |
| Wang Y, et al. 2024 ^48 update^ | UK | A prospective cohort study using data from the UK Biobank | Nationwide | 26101 | 68.5 | 44.3% | First positive COVID-19 test | Mixed IP/OP/ICU | 12 months | NM | Depression (14%)  Anxiety (14.3%) | Higher risk for those hospitalized, Lower risk for fully vaccinated individuals | Hospitalization |
| Whiteside DM, et al. 2023 ^680^ | USA | cross-sectional  study | Single center | 43 | 48.6 (12.6) | 16.3 | Date of the first positive COVID test | Mixed IP/OP/ICU | 6 months | Personality Assessment Inventory (PAI) | Depression (48.8%)  Anxiety (25.5%) | NM | - |
| Wong AW, et al. 2023 ^684^ | Canada | longitudinal cohort study | Multi center | 1344 | 51 (15) | 42% | Date of the first positive COVID test | Mixed IP/OP/ICU | 3-6 months | The EuroQol 5-Dimensions (EQ5D) | Depression (38%) | NM | - |
| Zhao Y, et al. 2021 ^1003^ | China | prospective cohort study | Multicenter | 94 | 48.11 | 58% | After hospital discharge | IP/ ICU | One year | 24 items, Hamilton Depression Rating Scale (HAMD-24) | Depression (42.5%) | Age, Pulmonary structural abnormalities and pulmonary diffusion capacities | - |
| Zhang L, et al. 2022 ^707^ | China | Cross-sectional Study | Single center | 255 | 43.78 (16.0) | 50.9% | After hospital discharge | IP/ ICU | 1 year | The EuroQol 5-Dimensions (EQ5D) | Depression and/or Anxiety (31.8%) | Older age, Number of post-COVID-19 symptoms | Older age, Number of post-COVID-19 symptoms |
| * It could include depression, anxiety, or suicidality  IP, inpatient;OP,outpatient;ICU,intensivecareunit;EMR,electronicmedicalrecords.  **IQR | | | | | | | | | | | | | |
